# Discovery of confined two-dimensional Laves tiling in a magnesium alloy

**DOI:** 10.1038/s41467-026-71932-9

**Published:** 2026-04-15

**Authors:** Hongbo Xie, Xiande Ma, Weixin Lou, Benfu Wang, Junpeng Li, Enyu Guo, Shanshan Li, Yiping Lu

**Affiliations:** 1 School of Materials Science and Engineering, Dalian University of Technology, Dalian, China; 2 Central Hospital of Dalian University of Technology, Dalian, China; 3 Instrumental Analysis Center, Dalian University of Technology, Dalian, China; 4 School of Materials Science and Engineering, Northeastern University, Shenyang, China; 5 Institute for Strategic Materials and Components, Shenyang University of Chemical Technology, Shenyang, China

**Keywords:** Metals and alloys, Condensed-matter physics

## Abstract

Laves phases composed of two-dimensional (2D) Laves tilings are prevalent in condensed matter systems, yet the existence of these 2D tilings as isolated structural motifs has remained unverified. Here, we report the discovery of a metastable, confined 2D Laves tiling precipitate in a compositionally dilute Mg alloy, revealed through aberration-corrected high-angle annular dark-field scanning transmission electron microscopy (HAADF-STEM) in combination with atomic-resolution energy-dispersive X-ray spectroscopy (EDS) mapping. The structure adopts a Laves-type Al_2_Ca configuration confined to the (0001) basal plane of the Mg matrix, with a thickness of merely a single tiling layer (~4.62 Å), stabilized by adjacent Mg/Ca interfacial atomic layers. Theoretical calculations reveal that tensile strain in the surrounding matrix suppresses the diffusion of small-sized Al solutes, thereby inhibiting out-of-plane coarsening. Notably, prolonged thermal exposure leads to the formation of multi-configurational layered Laves structures assembled from these fundamental units. These findings elucidate the precipitation behavior in Mg–Al–Ca alloys and uncover a structurally confined Laves tiling motif, advancing our understanding of Laves phase formation and solute clustering in metallic systems.

## Abstract (web summary)

A confined two-dimensional Laves unit is isolated and identified in an Mg alloy, which is only one unit-cell thick, revealing how interfacial strain limits its growth and providing new insight into the formation and stability of Laves phases.

## Introduction

Laves phases, a distinct subclass of Frank–Kasper structures, are broadly encountered in metallic systems, soft matter, self-assembled materials, and colloidal nanoparticles^1–14^. Their presence plays a critical role in governing both the mechanical and functional properties of materials. In structural metals, for example, Laves phases typically precipitate as hard intermetallic compounds, significantly enhancing yield strength and hardness^15^. Conversely, their accumulation at grain boundaries can lead to catastrophic embrittlement^16^. Beyond metallurgy, Laves architectures are increasingly explored for applications in photonic crystals, mechanical metamaterials, and self-healing materials^17,18^.

**Fig. 1:**
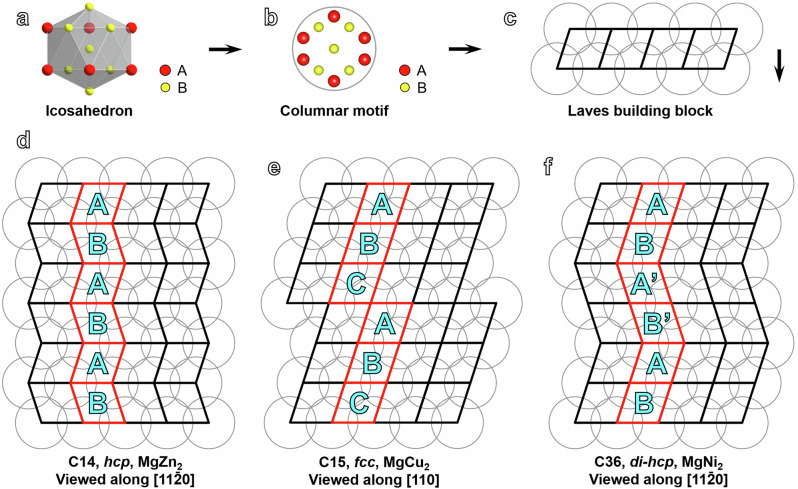
Structural motifs and crystallographic models of Laves phases. **a** Three-dimensional atomic model of an icosahedron in AB_2_-type Laves phases. **b** Columnar motif formed by the periodic stacking of icosahedra. **c** Laves building block constructed by periodic tiling of columnar motifs. **d–f** Crystallographic models of the three canonical Laves polytypes: **d** C14-type (hexagonal, *P6₃/mmc*); **e** C15-type (cubic, *Fd*$\bar{{{{\boldsymbol{3}}}}}{{{\boldsymbol{m}}}}$); and **f** C36-type (double hexagonal, *P6₃/mmc*), each comprising stacked Laves building blocks.

To date, more than 1400 distinct AB_2_-type Laves phases—where A and B represent atoms of larger and smaller radii, respectively—have been experimentally identified^1,2^. This number has continued to grow over the past three decades, aided by advances in high-throughput computational prediction methods. Crystallographically, Laves phases are typically classified into three principal polytypes^1,2,19^: (i) The MgZn₂-type (Fig. 1d), which crystallizes in a hexagonal close-packed (HCP) structure (space group *P6₃/mmc*) with an…ABAB… stacking sequence. Designated as the C14 Laves phase (the 14th complex structure identified), it remains the most common form; (ii) The MgCu₂-type (Fig. 1e), which adopts a face-centered cubic (FCC) structure (space group *Fd*$\bar{{{{\boldsymbol{3}}}}}{{{\boldsymbol{m}}}}$) with an…ABCABC… stacking sequence. This C15 Laves phase exhibits the highest symmetry and densest atomic coordination among the three; (iii) The MgNi₂-type (Fig. 1f), a C36 Laves phase with a double-HCP structure (space group *P6₃/mmc*), characterized by an…ABA′B′ABA′B′… stacking sequence. Unlike C14, the C36 structure requires two fundamental Laves building blocks to complete a full stacking unit. In sharp contrast to conventional FCC and HCP structures, where single atomic layers constitute the basic stacking units, Laves phases are assembled from multi-layered Laves tilings, termed Laves building blocks (Fig. 1c). Rather than originating from simple monolayer atomic arrangements, these blocks are geometrically derived from the periodic tiling of icosahedron columns (Fig. 1a, b), which define the underlying structural framework shared by all known Laves architectures.

The advent of aberration-corrected TEM, particularly HAADF-STEM, has enabled direct atomic-scale visualization of these topologically close-packed structures^3–6,9,18,20–27^. This capability has prompted a reassessment of the classical C14, C15, and C36 phases, which were long considered the only energetically favorable Laves configurations. Recent studies have revealed a much broader structural diversity built upon Laves building blocks, including non-periodic arrangements and even quasicrystalline structures featuring fivefold rotational symmetry^28–30^. Remarkably, whether in the classical polytypes, newly discovered layered configurations, or quasiperiodic structures, a universal architectural motif persists: all are fundamentally composed of rhombic layers with internal angles near 72°, formed by aligned icosahedron columns (Fig. 1c). This recurring architecture confirms the Laves building block as the fundamental structural unit, critical for understanding polymorphism and formation mechanisms in Laves-type phases. However, despite its central role, a stable, atomically thin monolayer variant has not yet been experimentally verified—representing a key gap in our structural knowledge.

Here, we show the direct observation of a confined, monolayer-thick Laves building block embedded within the (0001) basal plane of a dilute Mg alloy containing Al and Ca solute atoms, achieved via aberration-corrected HAADF-STEM combined with atomic-resolution EDS mapping and theoretical calculations. We identify a metastable nanoscale Laves lamella, whose precipitation strongly correlates with enhanced strength and hardness in the alloy. This finding provides the first atomic-scale evidence of an isolated Laves tiling and offers new insights into the nucleation, stability, and stacking behavior of Laves-type phases in complex condensed matter systems.

## Results and discussion

The age-hardening response of the solution-treated Mg–2.0 wt% Al–1.0 wt% Ca alloy, subjected to isothermal aging at 200 °C, is presented in Supplementary Fig. 1. The Vickers hardness increases substantially from ~43 HV to a peak of ~66 HV, followed by a gradual decline with extended aging. To elucidate the origin of this hardness evolution, we carried out systematic microstructural analyses at selected aging intervals.

**Fig. 2:**
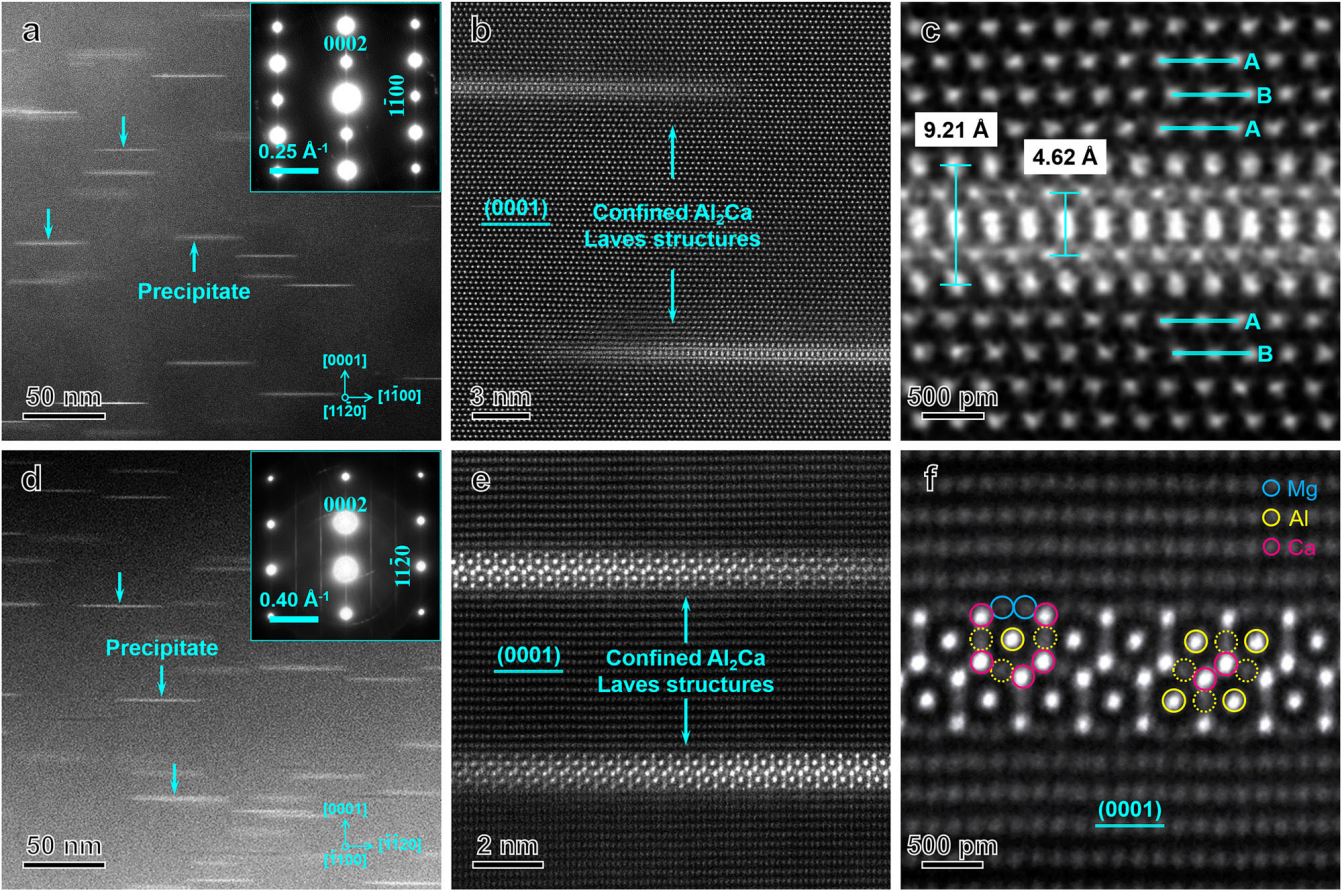
HAADF-STEM images reveal the presence of confined 2D Laves precipitates in the Mg–2.0Al–1.0Ca alloy aged at 200 °C for 24 h. HAADF-STEM images acquired along two orthogonal zone axes, **a–c** [11$\bar{2}0$]_α_, and **d**–**f** [1$\bar{1}00$]_α_, highlight the morphology and atomic arrangement of the precipitates. The corresponding SAED patterns are shown in the top-right insets of (**a** and **d**), respectively. Blue, yellow, and red circles in (**f**) represent Mg, Al, and Ca atoms, respectively.

HAADF-STEM images of the alloy aged for 24 h, viewed along the [11$\bar{{{{\bf{2}}}}}{{{\bf{0}}}}$]_α_ and [1$\bar{{{{\bf{1}}}}}{{{\bf{00}}}}$]_α_ zone axes, respectively, are shown in Fig. 2. Low-magnification images (Fig. 2a, d) reveal a high density of nanoscale, platelet-shaped precipitates uniformly distributed within the HCP matrix, with lateral dimensions of ~30–50 nm and thicknesses below 1 nm. Complementary bright-field TEM images are presented in Supplementary Fig. 2. The corresponding selected area electron diffraction (SAED) patterns, displayed in the top-right insets of Fig. 2a, d, exhibit pronounced streak-like features associated with the precipitates, reminiscent of those produced by stacking faults^31,32^. However, the absence of sharp diffraction spots renders direct structural determination from diffraction alone inconclusive.

To elucidate the atomic structure of the platelet-shaped precipitates, high-resolution STEM imaging was carried out. The precipitates in Fig. 2a, d are magnified, and the corresponding atomic-resolution HAADF-STEM images are shown in Fig. 2b, e. Both images clearly reveal two precipitate-nanoplates embedded within the (0001) basal plane of the Mg matrix.

A magnified region of Fig. 2b is further presented in Fig. 2c, providing atomic-scale insight into the internal structure of a representative nanoplate. Periodically arranged bright atomic columns are clearly visible within the precipitate, displaying a configuration closely resembling that of Laves-type structures^2^. However, the narrow interatomic spacing along the [11$\bar{2}0$]_α_ direction hinders full resolution of individual atomic columns. Precise measurements indicate the nanoplate exhibits a total thickness of 9.12 Å, contrasting with the 4.62 Å measured for the core region, which excludes the interfacial atomic layers. Importantly, the image in Fig. 2c also illustrates how the platelet-shaped precipitate is coherently embedded within the ABAB-stacked HCP Mg lattice. Analysis of multiple precipitates consistently shows an identical insertion sequence, in which the nanoplate is embedded between an ABA–precipitate–AB stacking sequence of the matrix, as annotated in cyan in Fig. 2c.

Figure 2f presents an atomic-scale HAADF-STEM image recorded along the [1$\bar{1}00$]_α_ zone axis, clearly demonstrating that the platelet-shaped precipitate adopts a structure related to the Laves phase. A cluster comprising ten atomic columns surrounding a central column—forming an icosahedral motif—is highlighted, corresponding to the columnar motif previously identified in Fig. 1b. A rhombic unit composed of four such columnar motifs, with internal angles close to 72°, is also outlined, representing a canonical structural feature of Laves phases. Structural analysis of all precipitates in the peak-aged alloy consistently reveals this confined, 2D Laves architecture, with a thickness equivalent to a single Laves building block, as illustrated in Fig. 1c.

**Fig. 3:**
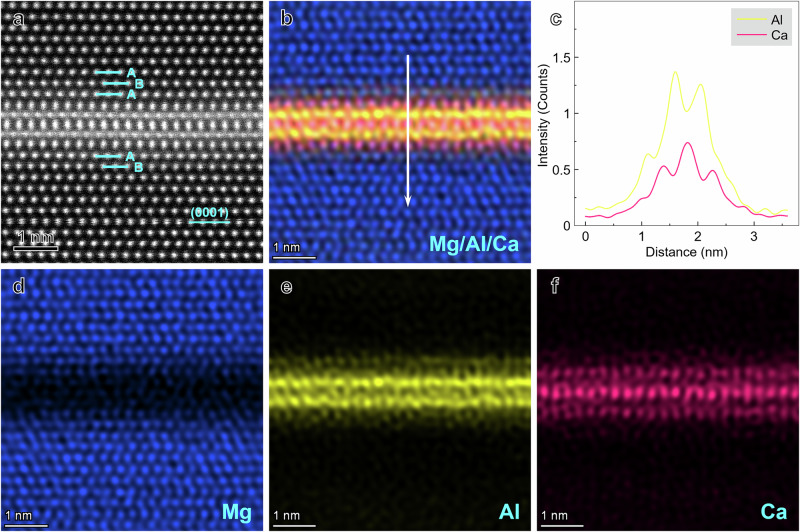
Atomic-scale HAADF-STEM imaging and EDS mapping of the peak-aged Mg–2.0Al–1.0Ca alloy. **a** HAADF-STEM image acquired along the [11$\bar{2}0$]_α_ zone axis; labels A and B indicate the characteristic alternating atomic stacking sequence in the Mg matrix. **b** Composite EDS elemental map (Mg–Al–Ca overlay) showing the spatial distribution of elements. The white line marks the position of the line scan across the precipitate. **c** Elemental intensity profiles of Al (yellow) and Ca (red) along the line scan in (**b**), revealing compositional variations across the confined 2D Laves precipitate. **d–f** Individual EDS elemental maps showing the distributions of **d** Mg (blu**e**); **e** Al (yellow); and **f** Ca (red). Source data are provided as a Source Data file.

In Fig. 2e, atomic sites within the precipitate are annotated solely based on the characteristic atomic arrangement of the Laves phase. Notably, the bright central column of the icosahedral motif is labeled as Al. While this assignment appears counterintuitive in the context of HAADF-STEM imaging—where contrast scales approximately with the square of the average atomic number (*Z*)—it is important to note that Al (*Z* = 13) is close in atomic number to Mg (*Z* = 12) and significantly lighter than Ca (*Z* = 20)^33^. As such, a prominent intensity corresponding to Al would not typically be expected in this system. To verify the atomic assignments, particularly at chemically critical lattice sites, we performed atomic-scale EDS mapping to determine the composition of the confined 2D Laves precipitate. The results, acquired along the [11$\bar{2}0$]_α_ zone axis, are shown in Fig. 3. The original HAADF-STEM image is presented in Fig. 3a, and the corresponding EDS elemental maps are displayed in Fig. 3b, d–f: a composite Mg–Al–Ca overlay (Fig. 3b), and individual elemental maps for Mg (Fig. 3d), Al (Fig. 3e), and Ca (Fig. 3f). These maps reveal that the confined 2D Laves precipitate is enriched in both Al and Ca, with the two elements distributed in an alternating, ordered fashion across specific atomic layers. A compositional line profile taken across the nanoplate—indicated by the white arrow in Fig. 3b and shown in Fig. 3c—confirms an approximate Al:Ca atomic ratio of 2:1 within the confined 2D Laves precipitate.

**Fig. 4:**
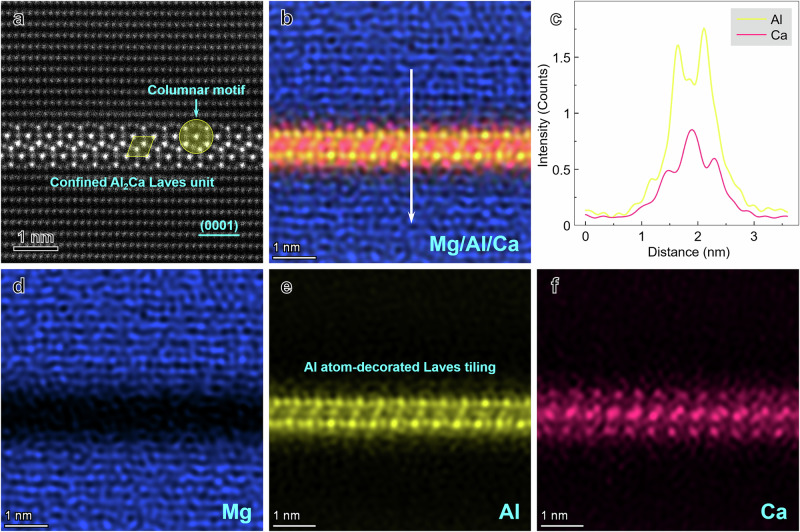
Atomic-scale HAADF-STEM imaging and EDS mapping of the peak-aged Mg–2.0Al–1.0Ca alloy. **a** HAADF-STEM image acquired along the [1$\bar{1}00$]_α_ zone axis. **b** Composite EDS elemental map (Mg–Al–Ca overlay) showing the spatial distribution of elements. The white line marks the position of the line scan across the precipitate. **c** Elemental intensity profiles of Al (yellow) and Ca (red) along the line scan in (**b**), revealing compositional variations across the confined 2D Laves precipitate. **d–f** Individual EDS elemental maps showing the distributions of **d** Mg (blu**e**); **e**, Al (yellow); and **f** Ca (red). Source data are provided as a Source Data file.

Atomic-scale EDS mapping results acquired along the [1$\bar{1}00$]_α_ zone axis is presented in Fig. 4. The corresponding compositional line profile (Fig. 4c) further confirms that the confined 2D Laves precipitate adopts a stoichiometry close to Al₂Ca. In determining this composition, signal contributions from Ca enrichment at the precipitate–matrix interfaces were excluded—a critical consideration discussed in detail below. As shown in Fig. 4e, the characteristic rhombic tiling of the Laves structural unit, delineated by Al atomic columns, is clearly resolved. Further structural analysis reveals that the pronounced HAADF contrast at the rhombic vertices—corresponding to the centers of the icosahedral motifs—originates from a local doubling of atomic column density relative to adjacent sites. Moreover, two atomic columns exhibiting markedly enhanced contrast within the rhombic tiling are identified as Ca (Fig. 4f). These Ca-enriched columns correspond to the A sites of the AB₂-type Laves phase, providing additional confirmation of the precipitate’s structural and compositional identity.

**Fig. 5:**
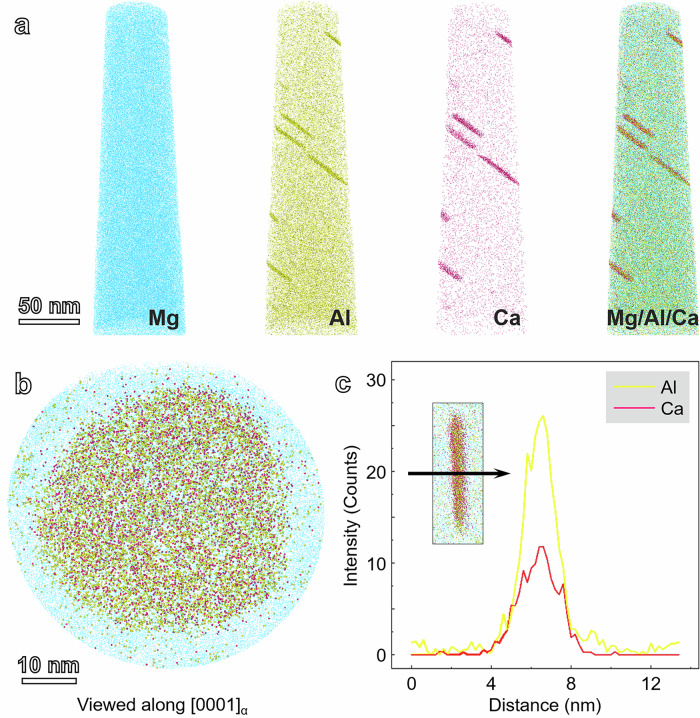
APT analysis of elemental distribution in the peak-aged Mg–2.0Al–1.0Ca alloy. **a** Three-dimensional atom maps showing the spatial distribution of Mg (blue), Al (yellow), and Ca (red), along with a composite overlay. **b** Corresponding atom distribution map viewed along the [0001]_α_ zone axis, highlighting the morphology of a confined 2D Laves precipitate. **c** Line-scan compositional profile extracted along the direction indicated by the black arrow in the top-left inset of (**c**), illustrating the elemental distribution across the 2D Laves precipitate. Source data are provided as a Source Data file.

Atom probe tomography (APT) analysis of the peak-aged Mg–2.0Al–1.0Ca alloy reveals the spatial distribution and composition of confined 2D Laves precipitates (Fig. 5). Three-dimensional atom maps (Fig. 5a) show that Al- and Ca-enriched precipitates are arranged in a parallel array within the Mg matrix, each extending ~30–50 nm in length—consistent with dimensions determined from HAADF-STEM imaging. A projection along the [0001]_α_ direction (Fig. 5b) illustrates the overall morphology of a representative nanoplate, which exhibits a nearly circular shape with a diameter of ~40 nm. A compositional profile taken normal to the platelet interface (Fig. 5c) confirms an internal Al:Ca atomic ratio close to 2:1, in excellent agreement with atomic-resolution EDS measurements.

**Fig. 6:**
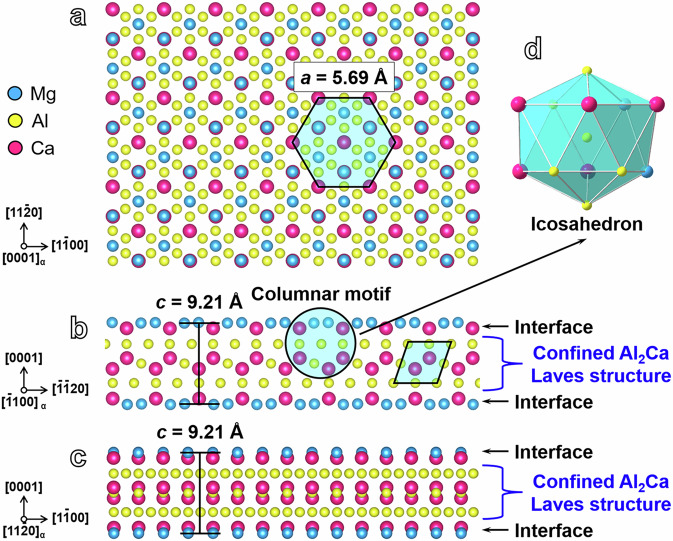
First-principles calculated atomic structure and columnar motif of the confined 2D Laves precipitate. **a–c** Atomic-resolution images projected along the **a** [0001]; **b** ${\left[\bar{1}100\right]}_{{{{\rm{\alpha }}}}}$; and **c** ${\left[11\bar{2}0\right]}_{{{{\rm{\alpha }}}}}$ zone axes, showing both in-plane and cross-sectional atomic arrangements. The regular hexagon in (**a**) reveals the sixfold rotational symmetry of the precipitate within the basal plane, with a lattice parameter of 5.69 Å. In (**b**), the rhombic and circular frames highlight the Laves building block and columnar motif, respectively, which represent the core structural features of the Laves phase. **d** Three-dimensional atomic model of an icosahedral cluster, highlighting the fundamental local motif. Blue, yellow, and red spheres represent Mg, Al, and Ca atoms, respectively. Source data are provided as a Source Data file.

Guided by atomic-scale HAADF-STEM imaging and compositional data from EDS and APT, the atomic configuration and cluster model of the confined 2D Laves precipitate were constructed via first-principles calculations, as illustrated in Fig. 6. The corresponding atomic-scale optimization process is provided in Supplementary Videos 1–3. The projection along the [0001]_α_ direction (Fig. 6a) reveals sixfold rotational symmetry within the basal plane, with an in-plane lattice parameter of approximately 5.69 Å. A view along the [$\bar{1}100$]_α_ direction (Fig. 6b) most clearly exposes the characteristic structural motif of the confined 2D Laves precipitate: a rhombic tiling (yellow rhombus) formed by the periodic arrangement of icosahedral clusters (columnar motif, yellow circle). As annotated on the left side of Fig. 6b, we define the region delineated by the rhombic tiling as the confined Al₂Ca Laves structure. This planar unit comprises only a single unit-cell thickness and deviates fundamentally from any of the three classical Laves phase prototypes. Rather than extending into a three-dimensional network, the structure is bounded above and below by interfacial atomic layers enriched in Mg and Ca. These layers are not merely peripheral boundaries but form an integral part of the precipitate: the interfacial atoms directly participate in the construction of the core icosahedral cluster—the fundamental motif of Laves-type phases—as illustrated by the yellow-circled columnar motif in Fig. 6b. Including these interfacial atoms in the structural model yields an overall composition of Mg₄Al₇Ca₈ for the confined precipitate. A three-dimensional atomic model of the constituent icosahedral cluster is shown in Fig. 6d, where a central Al atom is coordinated by twelve nearest neighbors—five Al, five Ca, and two Mg atoms—forming a complete icosahedron. The simulated HAADF-STEM image along [$\bar{1}100$]_α_ (Supplementary Fig. 3) shows excellent agreement with experimental observations. The projection along the [11$\bar{2}0$]_α_ direction (Fig. 6c) further reveals the densely packed atomic arrangement, which accounts for the inherent challenges in atomically resolving and chemically mapping such precipitate using current analytical techniques.

Computational analysis of the substitutional defect formation energies for Mg within this confined 2D Laves structure (Supplementary Fig. 4 and Supplementary Table 1) suggests that incorporation of Mg atoms beyond the interfacial layer is thermodynamically forbidden.

**Fig. 7:**
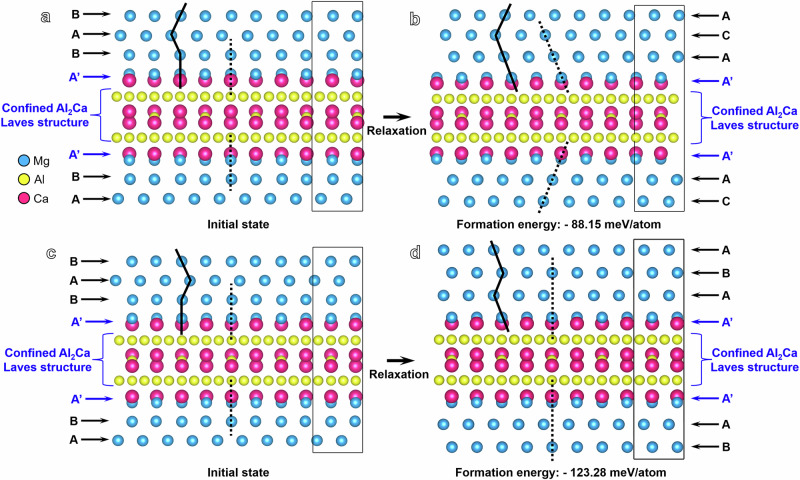
First-principles calculations of confined 2D Laves precipitates embedded in the Mg matrix. **a**, **c** Initial atomic configurations of the sandwiched 2D Laves precipitate, viewed along the ${\left[11\bar{2}0\right]}_{{{{\rm{\alpha }}}}}$ direction. **b**, **d** Fully relaxed structures after geometric optimization, converging to the energetically favorable ABAA′–A′AB stacking sequence, consistent with experimental observations. Blue, yellow, and red spheres represent Mg, Al, and Ca atoms, respectively. Source data are provided as a Source Data file.

First-principles calculations of the confined 2D Laves precipitates embedded in the Mg matrix are shown in Fig. 7. The initial structural models (Fig. 7a, c) align the outer interfacial layer of the precipitate (denoted A′) with the B-layer of the Mg matrix, but differ in the relative positioning of the A-layer: in Fig. 7a, the A-layer lies to the left of the B-layer, whereas in Fig. 7c, it lies to the right. The corresponding relaxed structures are presented in Fig. 7b, d. In Fig. 7b, geometric relaxation induces a relative displacement between the precipitate and matrix, resulting in a metastable configuration. As indicated by the dashed black lines, the A′AC atomic layers adopt an ABC stacking sequence reminiscent of FCC packing. However, this arrangement is energetically less favorable and has not been observed experimentally. By contrast, the configuration in Fig. 7d is in excellent agreement with experimental observations. Following relaxation, the precipitate and matrix shift such that the matrix B-layer aligns precisely with the interfacial A′-layer of the precipitate, as marked by the dashed black lines. This configuration is also more energetically favorable, with a formation energy of −123.28 meV per atom—substantially lower than that of the metastable structure in Fig. 7b (−88.15 meV per atom). Furthermore, the precipitate is fully coherent with the matrix along the (0001)_α_ basal plane.

**Fig. 8:**
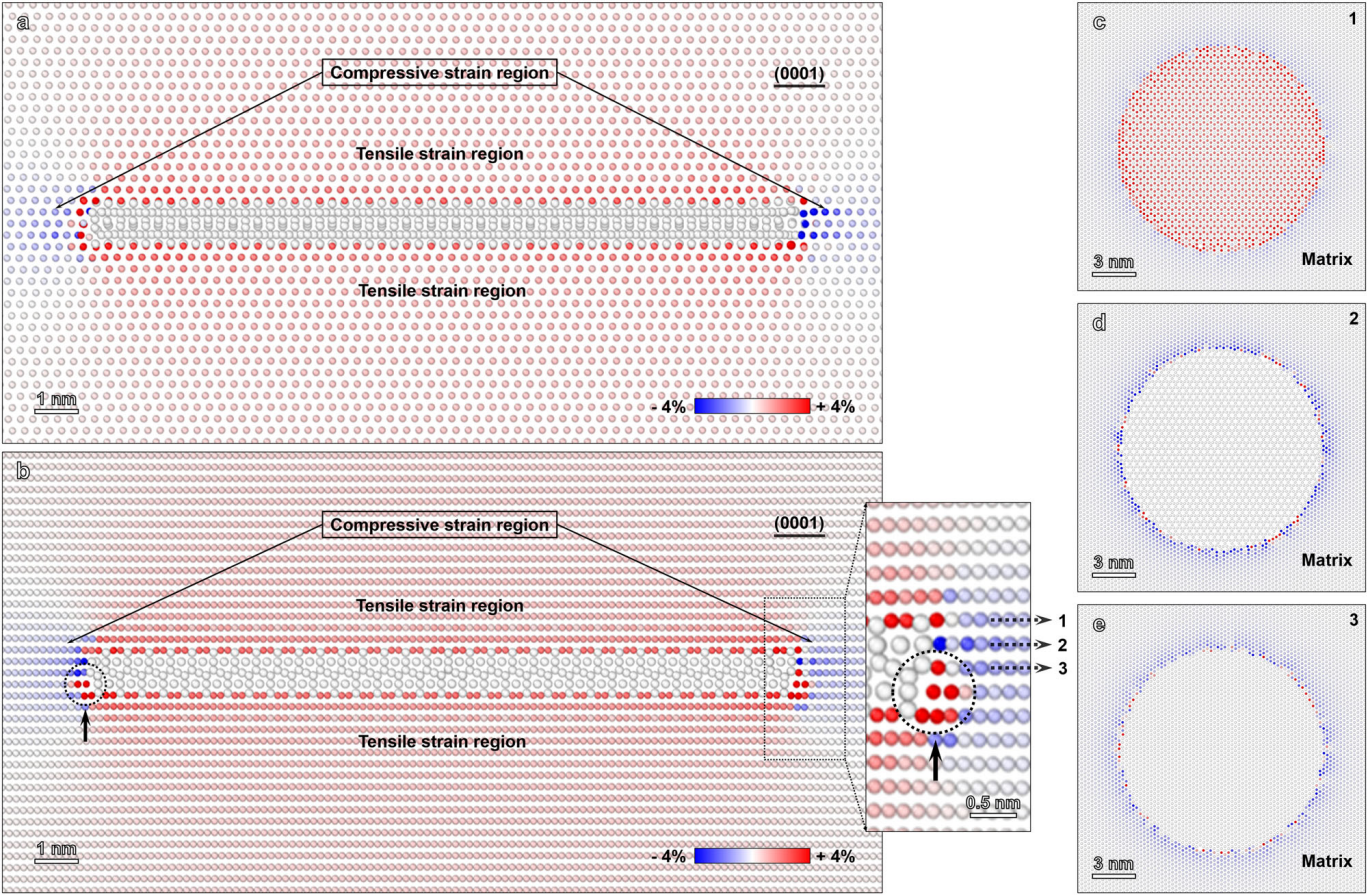
MD simulations and strain analysis of a confined 2D Laves precipitate embedded in the Mg matrix. **a**, **b** Atomic volume strain distributions obtained via Voronoi analysis, projected along the [11$\bar{2}0$]_α_ (**a**) and _[_10$\bar{1}0$]_α_ (**b**) directions. Tensile strain appears in basal atomic layers adjacent to the precipitate along [0001]ₐ, while alternating tensile and compressive zones are evident at the precipitate–matrix interface parallel to [0001]ₐ. The boxed region in (**b**) is enlarged to highlight local interfacial strain variations. **c–e** Layer-resolved strain profiles along [0001]ₐ for the three atomic layers marked as layers 1–3, revealing alternating strain concentrations across the interface. Source data are provided as a Source Data file.

Figure 8 presents molecular dynamics (MD) simulations of a confined 2D Laves precipitate embedded in the Mg matrix, combined with Voronoi analysis to evaluate the distribution of atomic volume strain. In these models, only the strain experienced by Mg atoms in the matrix is considered; strain within the precipitate itself is excluded from the analysis. The atomic volume strain maps projected along the [11$\bar{2}0$]_α_ and [10$\bar{1}0$]_α_ directions (Fig. 8a, b) reveal pronounced lattice distortions induced by the embedded precipitate. Red and blue denote tensile and compressive strain, respectively. Along the direction perpendicular to the platelet, i.e., the [0001]_α_ zone axis, basal plane Mg atoms adjacent to the precipitate exhibit tensile strain. In contrast, along the in-plane direction, alternating zones of compressive and tensile strain emerge at the precipitate–matrix interfaces. A similar interfacial strain distribution is confirmed at the atomic-scale by geometric phase analysis of HAADF-STEM images (Supplementary Fig. 5). These interfacial strain fields strongly influence solute behavior: tensile regions in the Mg matrix preferentially attract and facilitate the diffusion of large-sized solute atoms, whereas compressive zones favor the accumulation and migration of small-sized solutes—both scenarios being energetically favorable^34–36^. However, the distinct interfacial strain configuration surrounding the confined 2D Laves precipitate is energetically unfavorable for the diffusion or segregation of small-sized Al atoms along the [0001]_α_ direction. The calculated segregation energies for Al solutes at a series of lattice sites surrounding the confined Laves precipitate are shown in Supplementary Fig. 6. As a result, vertical thickening of the precipitate is suppressed, constraining its growth along the basal plane. An enlarged view of the boxed region in Fig. 8b reveals a strain-affected, partially formed icosahedral column at the matrix–precipitate boundary (outlined with a black dashed circle). Figure 8c–e present layer-resolved strain profiles along the [0001]_α_ direction for the three atomic layers labeled in Fig. 8b (layers 1–3). The strain profile in layer 1 (Fig. 8c) indicates excellent lattice matching between interfacial atoms and the adjacent Mg matrix, resulting in negligible misfit strain. By contrast, layers 2 and 3 exhibit significant lattice distortion, predominantly compressive in nature, due to mismatchs between the atomic planes of the precipitate and the matrix. Notably, the alternating tensile–compressive strain distribution across atomic layers may facilitate the selective diffusion and trapping of solute atoms, thereby promoting planar precipitate growth.

**Fig. 9:**
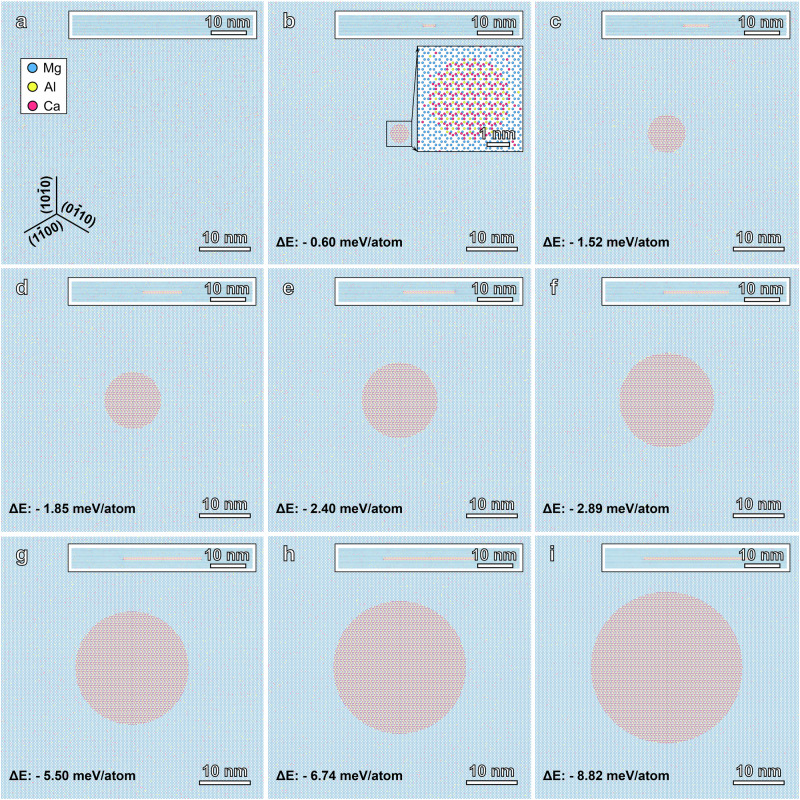
MD simulations of confined 2D Laves precipitate formation in the Mg matrix. **a** Atomic configuration of the initial solid-solution model viewed along the [0001]ₐ direction; with the corresponding [10$\bar{1}0$]_α_ projection shown in the top-right inset. **b–i** Simulated growth sequence of the confined 2D Laves precipitate, with each panel featuring a corresponding _[_10$\bar{1}0$]_α_ projection in the top-right corner. The inset in (**b**) provides a magnified view of the region indicated by the black box, highlighting the initial formation of the confined 2D Laves precipitate and the associated Al–Ca atomic ordering. The total system energy, displayed in the lower-left corner of each panel, decreases progressively as the precipitate grows, suggesting a thermodynamically favorable nucleation and evolution pathway. Blue, yellow, and red spheres represent Mg, Al, and Ca atoms, respectively. Source data are provided as a Source Data file.

Figure 9 presents MD simulations tracking the growth of confined 2D Laves precipitates within an Mg matrix. To ensure consistent energy comparisons, the total number of Mg, Al, and Ca atoms was held constant across all configurations. Figure 9a shows the atomic configuration of the initial solid-solution model, viewed along the [0001]ₐ direction, with the corresponding [10$\bar{1}0$]_α_ projection displayed in the top-right inset. In the subsequent simulations (Fig. 9b–i), the precipitate size was systematically increased to emulate its growth within the matrix. For each configuration, the total system energy was calculated and summarized in Supplementary Table 2, and the energy difference relative to the initial solid-solution state is indicated in the lower-left corner of each panel. As the precipitate enlarges, the relative energy decreases monotonically, indicating a progressive reduction in system energy. This energetically downhill trajectory suggests that the nucleation and growth of the confined 2D Laves precipitate are thermodynamically favorable, supporting its spontaneous formation within the matrix.

Our comprehensive microstructural characterization reveals that the confined 2D Al_2_Ca Laves structures exhibit remarkable stability within the Mg matrix, with no discernible phase transformation detected from the under-aged state (8 h, Supplementary Fig. 7) to pronounced over-ageing (200 h, Supplementary Fig. 8). The only change during this interval is a gradual lateral coarsening of the platelets, with their diameter increasing from an initial ~10 nm to a saturated size of ~50 nm. Under prolonged thermal exposure at elevated temperature, however, the monolayer Laves structure ultimately undergoes anisotropic thickening along the [0001]_α_ direction. HAADF-STEM imaging of the alloy aged at 200 °C for 500 h (Supplementary Figs. 9–11) reveals the emergence of two bilayer and two trilayer Laves polymorphic variants, assembled through the stacking of two and three Laves building blocks, respectively. Upon further aging to 800 h (Supplementary Fig. 12), these structures continue to thicken longitudinally. Notably, none of these configurations exhibit long-range periodicity and therefore cannot be classified within the three canonical Laves phase types. The emergence of these thickened polymorphs under over-aged condition highlights the thermodynamic metastability of the initially formed, single-unit-cell-thick 2D Al₂Ca Laves structure.

**Fig. 10:**
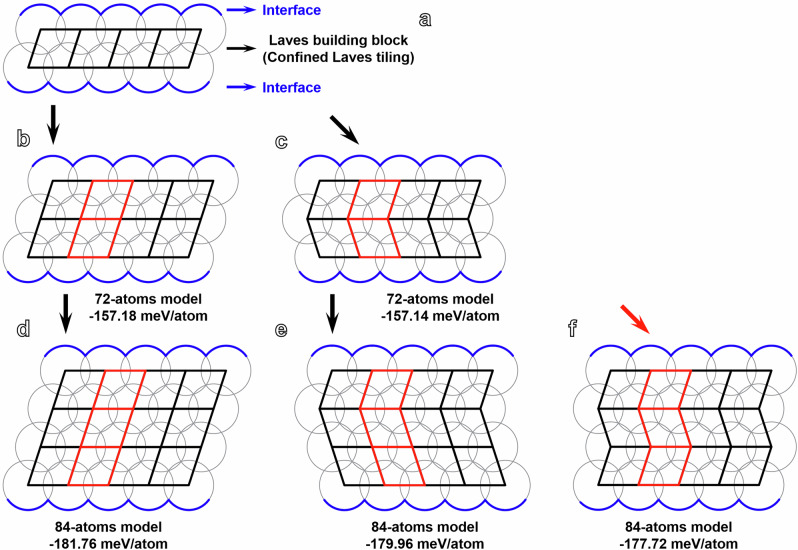
Crystallographic models of confined Laves polymorphs. **a** Laves building block formed by periodic tiling of columnar motifs (black circles). The black rhombi represent the individual unit cells of the confined 2D Laves phase. **b**, **c** Two experimentally confirmed polymorphic variants of the confined Laves phase, each composed of two stacked Laves building blocks. **d–f** Three structural variants comprising three stacked Laves building blocks. Of these, the configurations in (**d** and **e**) have been observed experimentally, whereas (**f**) remains unobserved, likely because of its comparatively higher formation energy. In each model, a single Al₂Ca Laves building block is outlined in red to highlight differences in stacking sequence and symmetry among the polymorphs. Blue lines denote the interfaces between the precipitates and the Mg matrix. Source data are provided as a Source Data file.

AB_2_-type Laves structures—comprising densely packed large (A) and small (B) atoms—are ubiquitous across metallic systems, soft matter, self-assembled materials, and colloidal nanoparticles^3,5–14^. Their fundamental motif, a rhombic Laves tiling with an internal angle of ~72°, serves not only as the structural basis of classical Laves phases but also as a key building block in complex Frank–Kasper phases, including the μ-, I-, and X-phases^26,37–39^. This rhombic tiling further underpins nanoscale domains with fivefold rotational symmetry, where it functions as a critical tessellation unit^28–30^. Recent advances in aberration-corrected STEM have revealed an expanding diversity of Laves configurations, many of which depart from the periodic stacking sequences of the classical C14, C15, and C36 phase^4,23,30,40–50^. These findings point to a far more polymorphic and structurally versatile Laves landscape than previously recognized. The polymorphic Laves configurations identified in this study are catalogued in Fig. 10. Figure 10b, c show two experimentally confirmed variants constructed from double-layer Laves building blocks, corresponding to Supplementary Fig. 10a and c, respectively. Figure 10d, e display three-layer configurations formed by stacking three Laves units (Supplementary Fig. 11a and c). Figure 10f illustrates a multi-bent three-layer variant that remains unobserved experimentally; First-principles calculations indicate that its higher formation energy—provided beneath each graph—may explain its absence. These polymorphic configurations, each assembled from fundamental Laves building blocks, underscore the extensive configurational diversity of the Laves phase family. The confined 2D Laves tiling unit reported here emerges as a unifying structural archetype among these complex configurations, offering critical insights into their nucleation pathways and growth mechanisms. This model provides a conceptual framework for understanding the formation of topologically close-packed phases and structurally complex aperiodic assemblies in metallic systems.

Mg–Al–Ca alloys are a class of ultralight structural materials with significant potential for aerospace, automotive, and electronic applications^51–55^. Despite their technological promise, the microstructural origins of their age-hardening behavior remain poorly understood. Classical models propose a transformation sequence during early-stage aging, beginning with the formation of an ordered Guinier–Preston (GP) zone, followed by precipitation of an FCC C15-type Al₂Ca Laves phase^56^. However, neither the proposed GP zone nor the C15-type structure has been directly observed via atomic-resolution STEM imaging. Here we present atomic-resolution evidence demonstrating that the initial precipitates adopts a confined 2D Al₂Ca Laves structure, just one unit-cell thick (~4.62 Å) along the [0001]_α_ direction. This initial configuration is structurally distinct from the host Mg matrix. With prolonged thermal exposure, these monolayer units thicken via sequential stacking, giving rise to a series of polymorphic Laves variants rather than evolving into a single C15-type Laves configuration. Precipitation in Mg–Al–Ca alloys not only impedes dislocation motion to enhance hardness but is also noted for achieving simultaneous strength and ductility—an uncommon attribute among Laves phase–strengthened materials^57,58^. Moreover, the resulting basal plane precipitates confer exceptional creep resistance^59^, underscoring their scientific and technological significance. Collectively, our findings revise the prevailing paradigm of phase evolution in Mg–Al–Ca alloys and provide mechanistic insights critical for the predictive design of next-generation lightweight structural materials.

In summary, we report the discovery of a confined 2D Laves tiling precipitate in a compositionally dilute Mg–Al–Ca ternary alloy. The embedded 2D Al₂Ca Laves structure adopts a well-ordered hexagonal arrangement (a = 5.69 Å) with a single unit-cell thickness along the $\left[0001\right]$_α_ direction (c = 4.62 Å). Atomic-scale stabilization is achieved through interfacial interactions with adjacent Mg/Ca atomic layers, which give rise to a distinctive …ABA′–Laves tiling–A′BA… stacking sequence along the [0001]_α_ crystallographic axis. Theoretical calculations reveal that tensile strain in the surrounding matrix inhibits out-of-plane growth by restricting the diffusion of small-radius Al solutes. While structurally stable over extended periods, this monolayer Laves structure is thermodynamically metastable. Upon prolonged isothermal aging, it evolves into a family of polymorphic multilayered variants through successive stacking of fundamental Laves building blocks. These findings uncover a structurally confined Laves tiling motif and provide critical insight into the nucleation, stabilization, and transformation of Laves structures, bridging classical Laves phases with their nanoscale counterparts. Furthermore, they offer theoretical guidance for the rational design of next-generation high-strength alloys reinforced by confined 2D Laves precipitates.

## Methods

### Alloy synthesis and microstructural characterization

An alloy with the nominal composition Mg–2.0 wt% Al–1.0 wt% Ca was synthesized by induction melting high-purity elemental Mg (99.9 wt%), Al (99.9 wt%), and Ca (99.9 wt%) under an argon atmosphere (50 Pa). The raw materials were obtained from Baowu Magnesium Technology Co., Ltd., China. The melt was maintained at 760 °C for 5 min with continuous stirring and then cast into a steel mold preheated to 300 °C. The as-cast ingots were solution-treated at 520 °C for 12 h, water quenched, and subsequently aged isothermally in an oil bath at 200 °C for various durations. Vickers hardness was measured using an HVS-50Z tester under a 2 kgf load with a 15 s dwell time. Thin foils for TEM/STEM were prepared by twin-jet electropolishing 3 mm discs at −40 °C in an electrolyte consisting of 5.3 g lithium chloride, 11.2 g magnesium perchlorate, 500 mL methanol, and 100 mL 2-butoxyethanol. Final thinning was achieved via low-energy ion milling (Fischione M1061), followed by plasma cleaning using a Gatan Solarus II system. TEM and STEM observations were performed on a ThermoFisher Spectra Ultra microscope operated at 300 kV, equipped with image/probe aberration correctors and an Ultra-X EDX spectroscopy system. TEM, STEM, and EDS mapping images were processed using Velox software (Velox 3.12), and no filtering was applied to any image. APT specimens were fabricated by focused ion beam (FIB, ThermoFisher Helios 5 Hydra UX) milling using a Xe-ion source, yielding tip radii of ~50 nm. APT analyses were conducted on a LEAP™ 6000 XR system in voltage-pulsing mode at 25 K, with a pulse fraction of 15% and a detection rate of 0.002 ions per pulse.

### First-principles calculations

The energetics of confined Laves units embedded in the Mg matrix were evaluated using density functional theory (DFT), as implemented in the Vienna ab initio Simulation Package (VASP, version 5.4.4)^60^. Electron–ion interactions were described using the projector augmented-wave (PAW) method^61,62^, and exchange–correlation effects were treated with the Perdew–Burke–Ernzerhof (PBE) functional within the generalized gradient approximation (GGA)^63^.

Calculations employed a plane-wave energy cutoff of 520 eV and a Γ-centered Monkhorst–Pack k-point mesh. Structural relaxations were performed using the conjugate gradient method with a force convergence criterion of 0.01 eV Å^−1^. A two-step relaxation protocol was used: variable-cell optimization (converged to 10^−5^ eV) followed by fixed-volume calculations (converged to 10^−7^ eV) for accurate total energy determination.

The formation energy (${E}_{f}$) of the embedded Al₂Ca Laves structures was calculated as:

1 $${E}_{{{{\rm{f}}}}}=\frac{{{{\boldsymbol{E}}}}\left({{{{\bf{Mg}}}}}_{{{{\bf{x}}}}}{{{{\bf{Al}}}}}_{{{{\bf{y}}}}}{{{{\bf{Ca}}}}}_{{{{\bf{z}}}}}\right)-{{{\boldsymbol{E}}}}({{{{\bf{Mg}}}}}_{{{{\bf{x}}}}})-{{{\boldsymbol{E}}}}\left({{{{\bf{Al}}}}}_{{{{\bf{y}}}}}\right)-{{{\boldsymbol{E}}}}\left({{{{\bf{Ca}}}}}_{{{{\bf{z}}}}}\right)}{{{{\boldsymbol{x}}}}+{{{\boldsymbol{y}}}}+{{{\boldsymbol{z}}}}}$$

where $E({{{{\rm{Mg}}}}}_{{{{\rm{x}}}}}{{{{\rm{Al}}}}}_{{{{\rm{y}}}}}{{{{\rm{Ca}}}}}_{{{{\rm{z}}}}})$ is the total energy of the Mg supercell containing Al_2_Ca Laves structures, and $E({{{{\rm{Mg}}}}}_{{{{\rm{x}}}}})$, $E({{{{\rm{Al}}}}}_{{{{\rm{y}}}}})$, and *E*(Ca_z_) are the energies of pure Mg, Al, and Ca supercells containing *x*, *y*, and *z* atoms, respectively^64^.

### Molecular dynamics simulations

Molecular dynamics (MD) simulations were carried out using the LAMMPS software package (version 29 Oct 2020)^65^, with interatomic interactions modeled via a modified embedded-atom method (MEAM) potential optimized for the Mg–Al–Ca ternary system^66^. Structural equilibrium was achieved through a multi-step relaxation process. The system was first equilibrated at 300 K for 100 ps using a Nosé–Hoover thermostat in the isothermal–isobaric (NPT) ensemble to eliminate residual stresses. This was followed by simulated annealing and conjugate gradient energy minimization to remove thermal vibrations, resulting in fully relaxed atomic configurations suitable for subsequent energy and strain analysis. The minimization was considered complete when either the total energy change between successive iterations was less than 10^−10^ (dimensionless) or the maximum force component on any single atom was less than 10^−10^ eV Å^−1^.

To probe the evolution of confined 2D Laves structures and the associated strain fields during precipitation, we employed a quasi-static virtual growth protocol. A disc-shaped cylindrical precipitate within the simulation cell was enlarged stepwise by iteratively replacing Mg atoms at the precipitate–matrix interface with Al or Ca solutes, with structural relaxation and energy minimization performed after each substitution.

## Supplementary information

Supplementary Information

Description of Additional Supplementary Files

Supplementary Video 1

Supplementary Video 2

Supplementary Video 3

Transparent Peer Review file

## Source data

Source Data

## Data Availability

The data generated in this study have been deposited in the public community repository. Figshare: 10.6084/m9.figshare.31518958. Source data are provided with this paper.
