# Supplementary material for: Discovery of confined two-dimensional Laves tiling in a magnesium alloy
Source: Nat Commun. 2026 Apr 15;17:5207. doi: 10.1038/s41467-026-71932-9 (PMC13253817; doi:10.1038/s41467-026-71932-9)
Supplement: Supplementary file 1 — Supplementary Information [file 41467_2026_71932_MOESM1_ESM.pdf]

Discovery of confined two-dimensional Laves tiling in a magnesium alloy

Supplementary Materials

Hongbo Xie[a, b, c], Xiande Ma[d] *, Weixin Lou[a], Benfu Wang[a], Junpeng Li[a], Enyu Guo[a], Shanshan Li[e] *, Yiping Lu[a] *

[a] *School of Materials Science and Engineering, Dalian University of Technology, Dalian, 116024, China*

[b] *Central Hospital of Dalian University of Technology, Dalian, 116024, China*

[c] *Instrumental Analysis Center, Dalian University of Technology, Dalian, 116024, China*

[d] *School of Materials Science and Engineering, Northeastern University, Shenyang 110819, China*

[e] *Institute Center for Strategic Materials and Components, Shenyang University of Chemical Technology, Shenyang, 110142, China*

* Corresponding authors. Email:

maxiande@gmail.com (X. Ma);

shanshanli_work@126.com (S. Li);

luyiping@dlut.edu.cn (Y. Lu).

Supplementary Methods

First-principles calculations

To systematically evaluate the thermodynamic stability of various site occupations, we calculated the substitutional defect formation energy E_{f} using the general formalism [1, 2]:

$$E_{\mathrm{f}} = E_{\mathrm{defect}} - E_{\mathrm{ref}} - n\,(\mu_{\mathrm{M}} - \mu_{\mathrm{H}})$$

where:

E_{defect} is the total energy of the supercell containing the substitutional defect.

E_{ref} is the total energy of the pristine, reference supercell.

n is the number of atoms being substituted.

μ_{M} is the chemical potential of the substituting element (the dopant/solute entering the lattice).

μ_{H} is the chemical potential of the host element (the atom being replaced at the specific lattice site).

In the specific context of the reviewer's question, M corresponds to Mg, and H corresponds to Ca/Al.

A positive value of E_{f} indicates that the substitution is thermodynamically unfavorable.

Geometric phase analysis

An open source software (Strain++), was used for geometric phase analysis of the atomic-scale HAADF-STEM images. A scale range of -5 % to +5 % was applied to the strain map.

Supplementary Figures

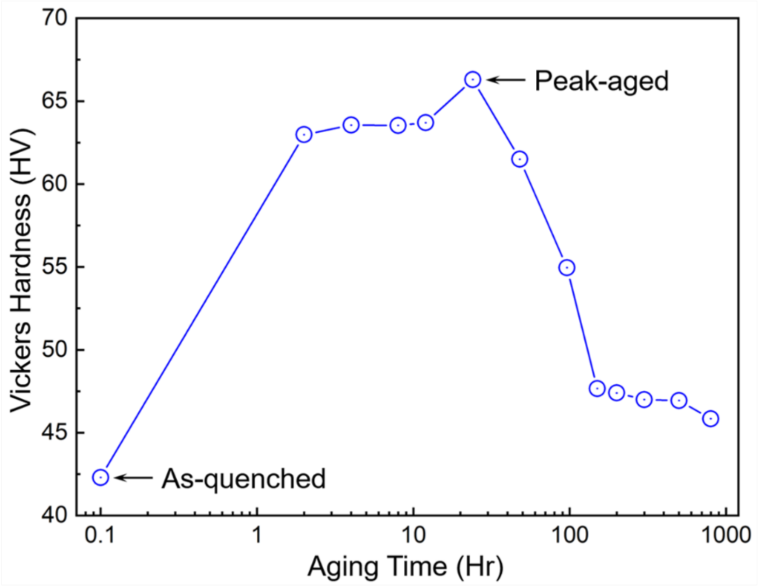

Supplementary Figure 1. Age-hardening response of the Mg–2.0Al–1.0Ca alloy. Vickers hardness evolution during isothermal aging at 200 °C, revealing the peak-aged condition. Source data are provided as a Source Data file.

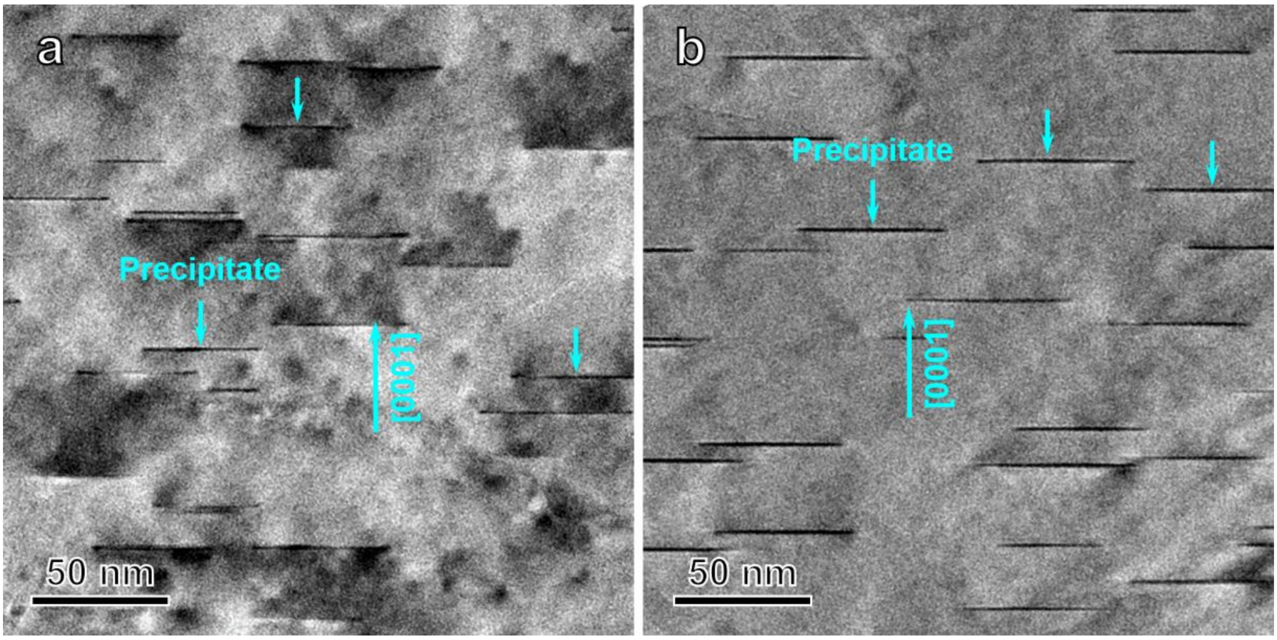

Supplementary Figure 2. Bright-field TEM images of confined two-dimensional Laves precipitates. a, **b**, TEM images acquired along the $[11\bar{2}0]_\alpha$ and $[1\bar{1}00]_\alpha$ zone axes, respectively, in the Mg–2.0Al–1.0Ca alloy aged at 200 °C for 24 h, showing the presence and morphology of confined 2D Laves precipitates.

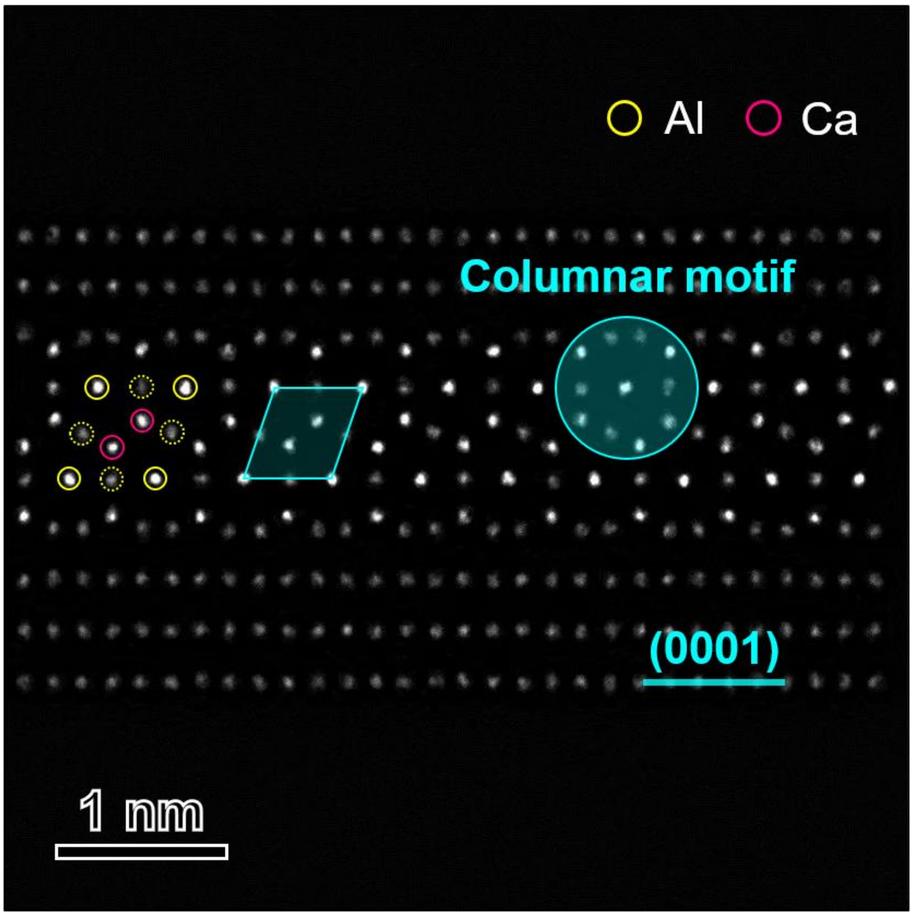

Supplementary Figure 3. Simulated HAADF-STEM image of a confined 2D Laves precipitate embedded in the Mg matrix. Multislice simulations reproducing the contrast features observed in experimental HAADF-STEM images.

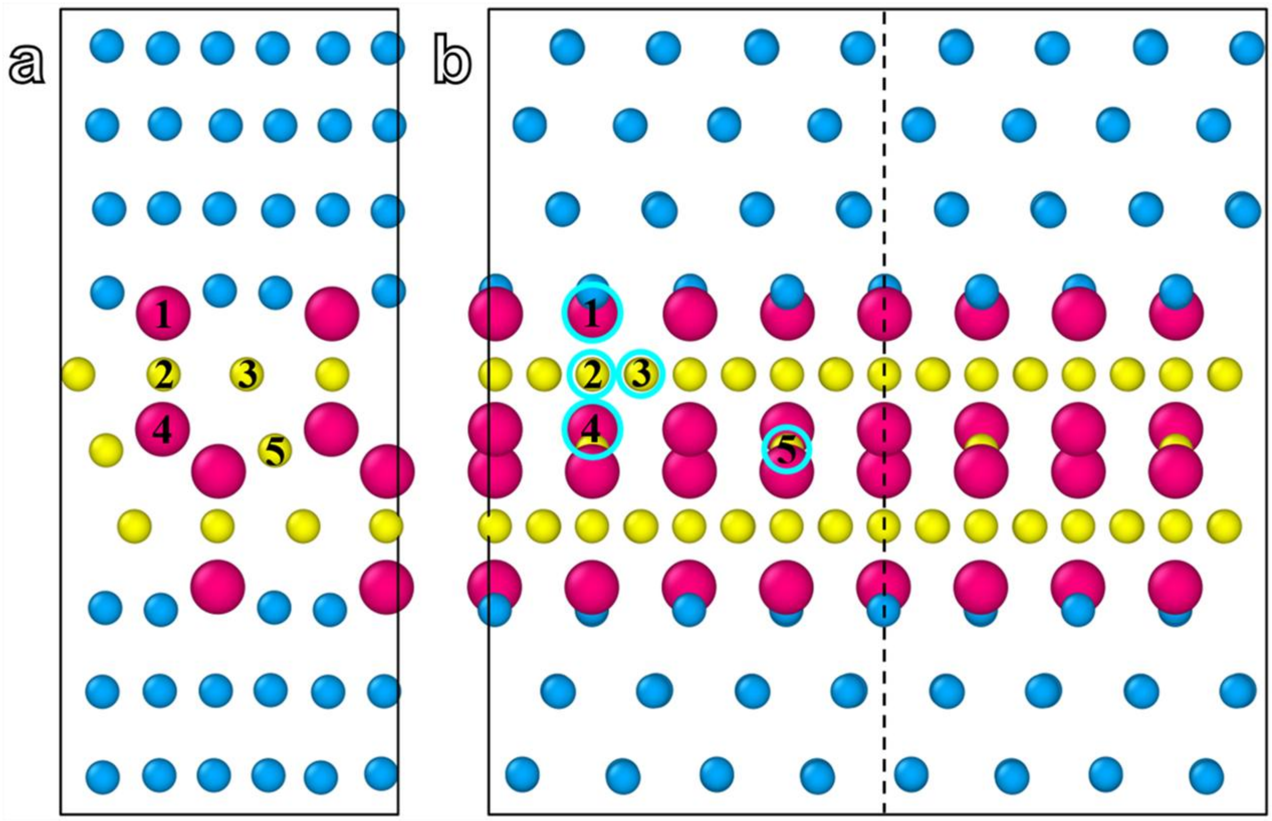

Supplementary Figure 4. Atomic models used to evaluate the thermodynamic stability of Mg atom substitution in the confined 2D Laves precipitate. Views along the $[\bar{1}100]_{\alpha}$ (**a**) and $[11\bar{2}0]_{\alpha}$ (**b**) zone axes. Source data are provided as a Source Data file.

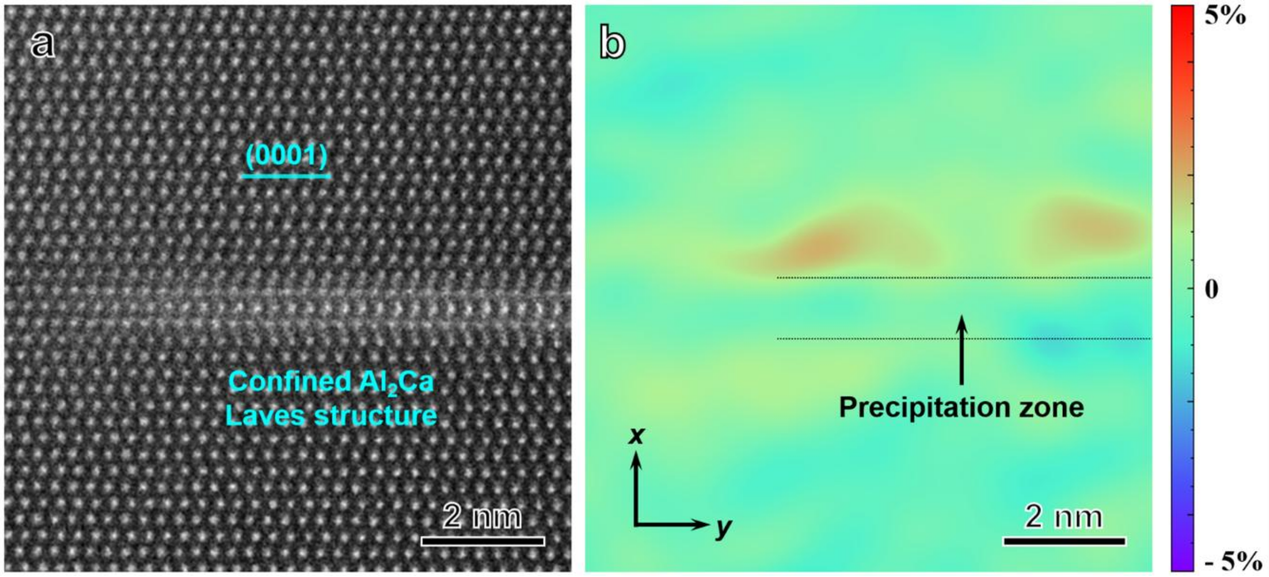

Supplementary Figure 5. Atomic-scale characterization of lattice strain. a, HAADF-STEM image acquired along the $[11\bar{2}0]_\alpha$ zone axis for the alloy aged at 200 °C for 24 h. **b**, Corresponding geometric phase analysis (GPA) map of the ε_{xx} strain component, measured normal to the (0001) basal plane.

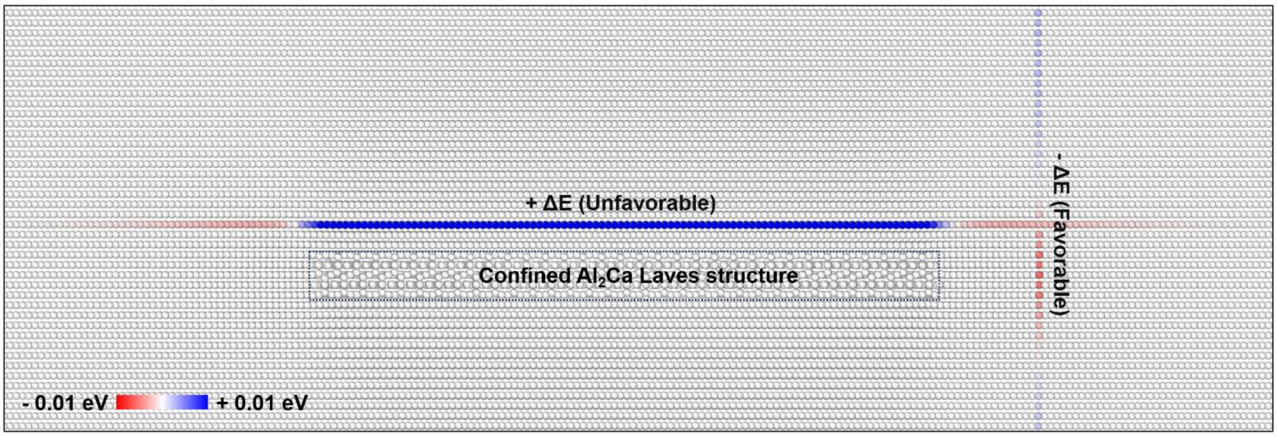

Supplementary Figure 6. Schematic illustration of the asymmetric energy landscape for Al-solute diffusion near the confined 2D Laves precipitate. The tensile strain field above the precipitate presents an energy barrier, whereas the lateral regions provide energetically favourable sites that facilitate solute diffusion. Source data are provided as a Source Data file.

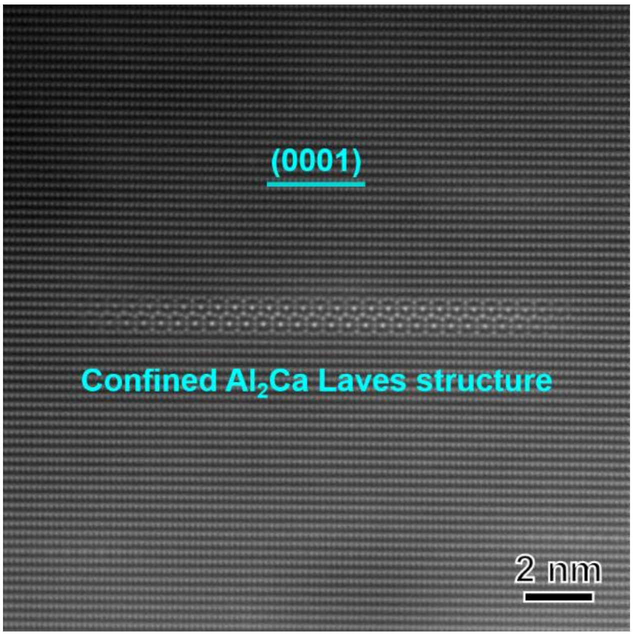

Supplementary Figure 7. HAADF-STEM image reveals the presence of a confined 2D Laves precipitate in the Mg–2.0Al–1.0Ca alloy aged at 200 °C for 8 h. The image acquired along the $[1\bar{1}00]_{\alpha}$ zone axis.

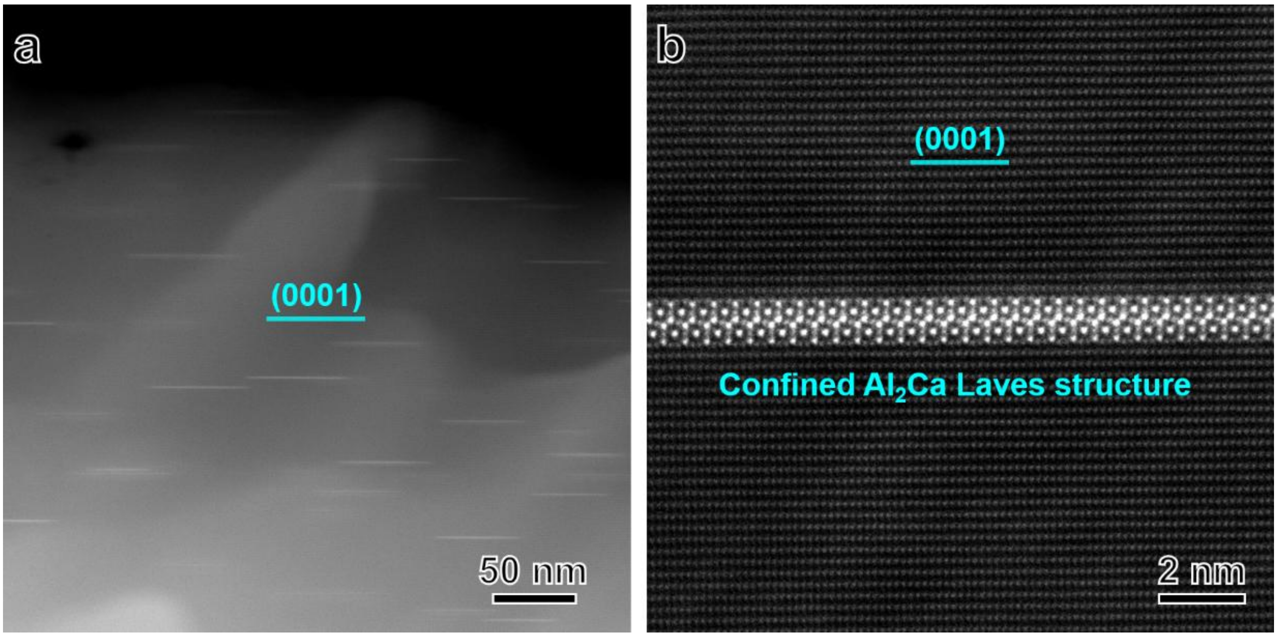

Supplementary Figure 8. HAADF-STEM images reveal the presence of confined 2D Laves precipitates in the Mg–2.0Al–1.0Ca alloy aged at 200 °C for 200 h. The images acquired along the $[1\bar{1}00]_{\alpha}$ zone axis.

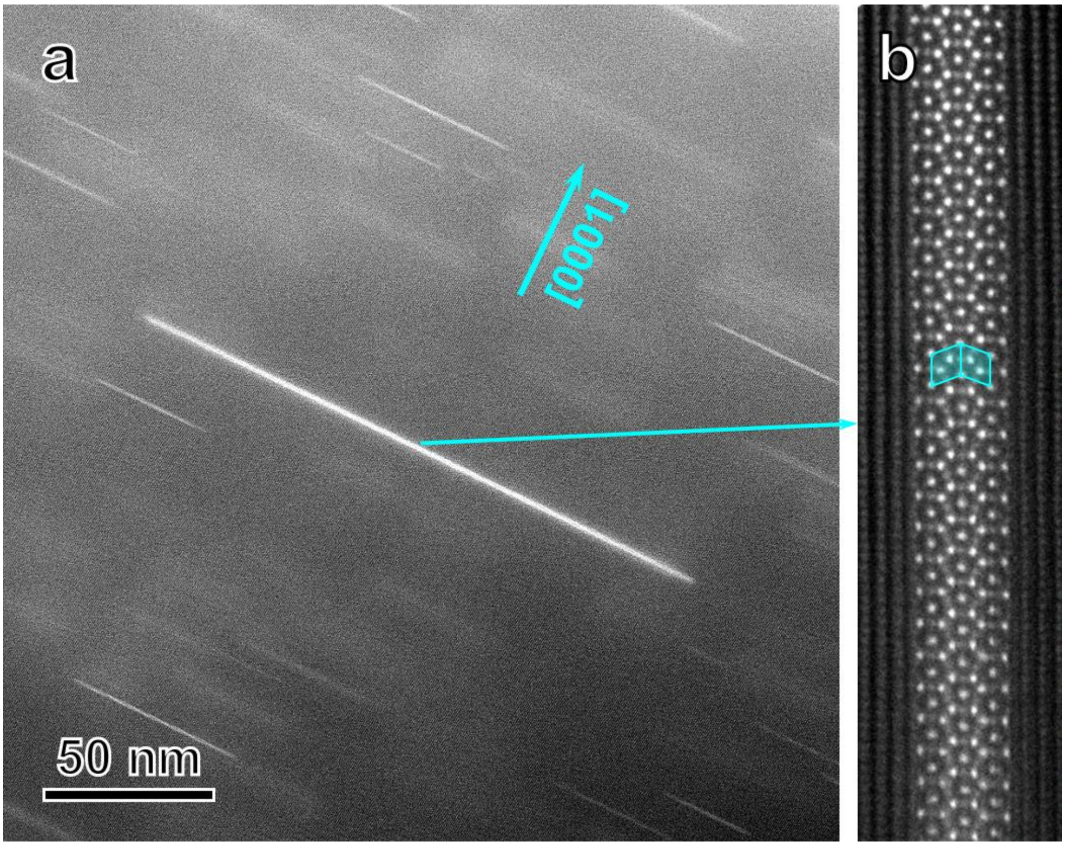

Supplementary Figure 9. HAADF-STEM images of the Mg–2.0Al–1.0Ca alloy aged at 200 °C for 500 h, acquired along the $[1\bar{1}00]_\alpha$ zone axis. a, A confined precipitate approximately 200 nm in lateral dimension is observed lying within the $(0001)_a$ basal plane. **b**, Magnified view of the region in ***a***, revealing a well-defined confined Laves phase composed of two stacked Laves building blocks.

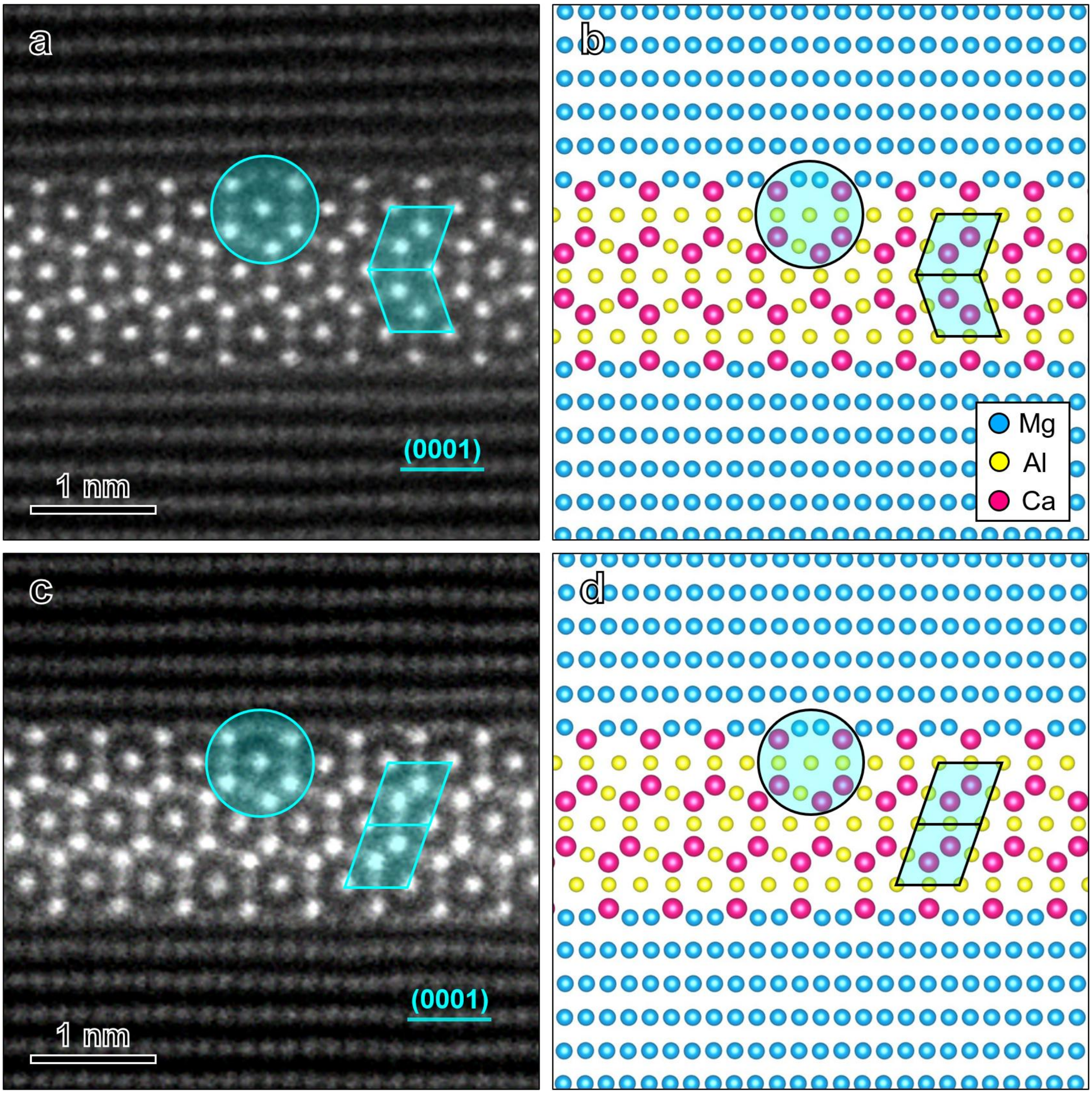

Supplementary Figure 10. Atomic-scale HAADF-STEM images and structural models of confined Laves polymorphs in the Mg–2.0Al–1.0Ca alloy aged at 200 °C for 500 h, viewed along the $[1\bar{1}00]_a$ zone axis. a, c, Atomic-resolution HAADF-STEM images revealing two distinct polymorphic variants of the confined Laves phase, each consisting of two stacked Laves building blocks. **b, d,** Corresponding atomic models obtained from MD simulations, showing the energetically optimized structures matching the observed configurations.

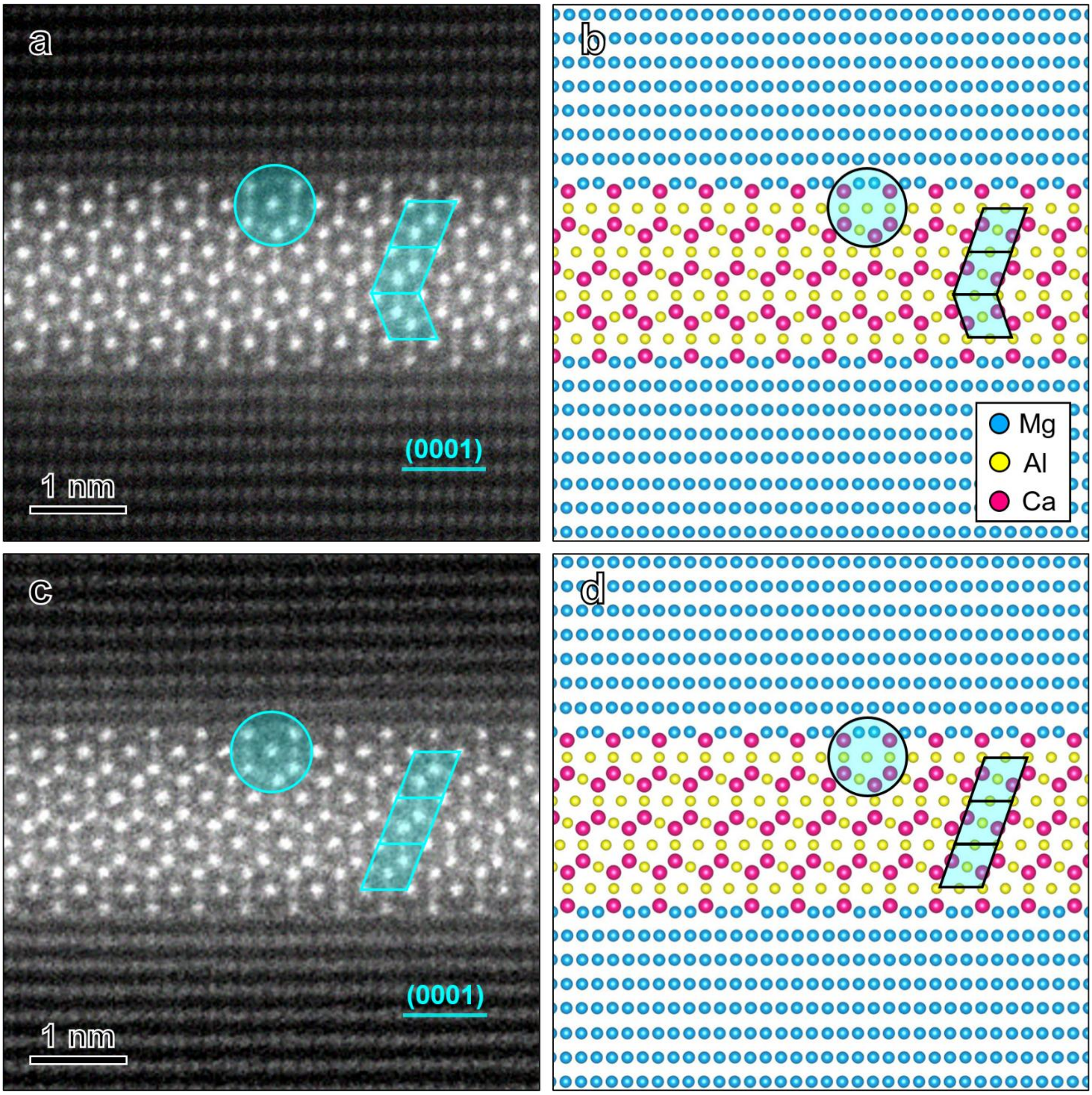

Supplementary Figure 11. Atomic-scale HAADF-STEM images and structural models of confined Laves polymorphs in the Mg–2.0Al–1.0Ca alloy aged at 200 °C for 500 h, viewed along the [1$\bar{1}$00]$_a$ zone axis. a, c, Atomic-resolution HAADF-STEM images revealing two distinct polymorphic variants of the confined Laves phase, each comprising three stacked Laves building blocks. **b, d,** Corresponding atomic models from MD simulations, illustrating the energetically favorable configurations associated with the observed structures.

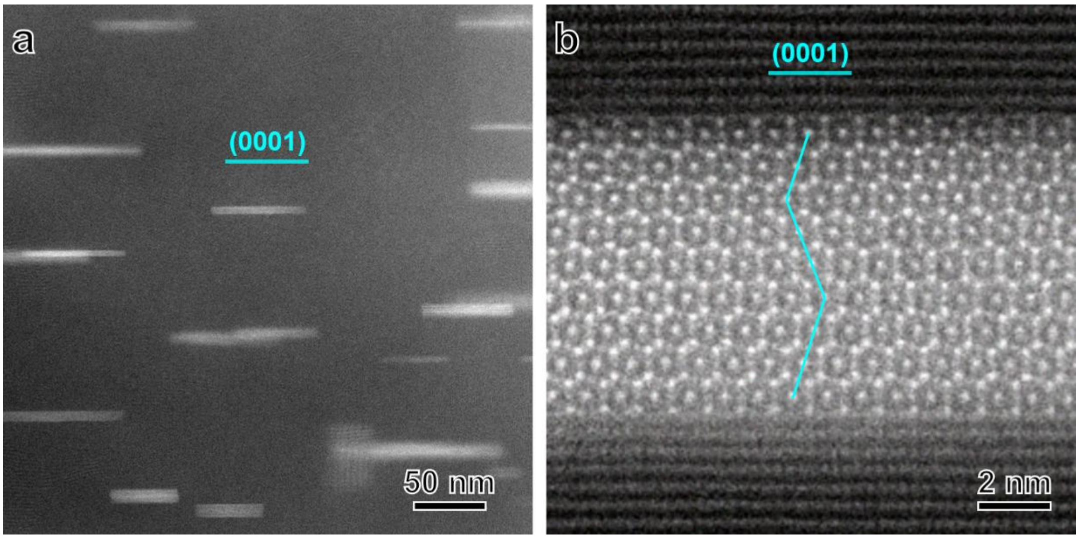

Supplementary Figure 12. HAADF-STEM images reveal the presence of Laves precipitates in the Mg–2.0Al–1.0Ca alloy aged at 200 °C for 800 h. The images acquired along the $[1\bar{1}00]_{\alpha}$ zone axis.

Supplementary Tables

Supplementary Table 1. Calculated formation energies of substitutional defects for Mg atoms at different lattice sites, demonstrating thermodynamic unfavorability. Source data are provided as a Source Data file.

	1	2	3	4	5
1*1*1	0.815 eV	0.509 eV	0.499 eV	0.912 eV	0.586 eV
1*2*1	0.780 eV	0.522 eV	0.633 eV	0.869 eV	0.593 eV

Supplementary Table 2. MD simulations of confined 2D Laves precipitate formation in the Mg matrix.

	Simulation model	Atom number in model	Total energy (eV)	ΔE (meV/atom)
**0**	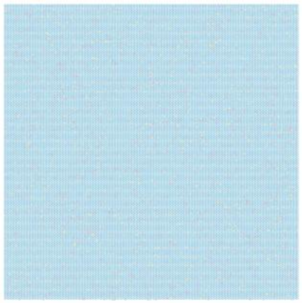	Mg: 771802<br>Al: 25980<br>Ca: 14810	-1308828.02	-
**1**	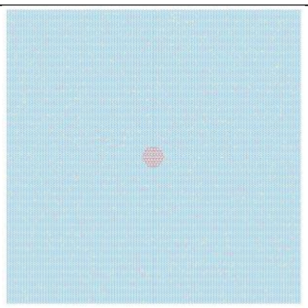	Mg: 771802<br>Al: 25980<br>Ca: 14810	-1309316.47	- 0.60
**2**	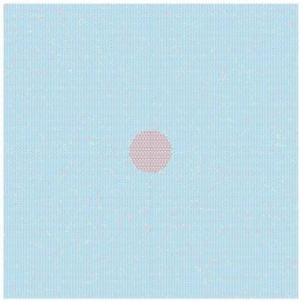	Mg: 771802<br>Al: 25980<br>Ca: 14810	-1310065.01	- 1.52
**3**	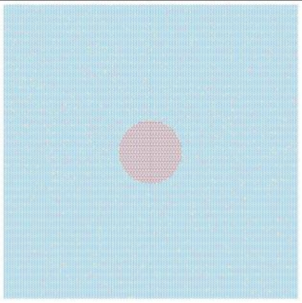	Mg: 771802<br>Al: 25980<br>Ca: 14810	-1310330.11	- 1.85
**4**	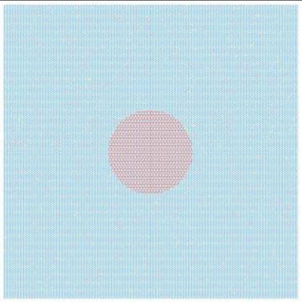	Mg: 771802<br>Al: 25980<br>Ca: 14810	-1310771.95	- 2.40

**5**	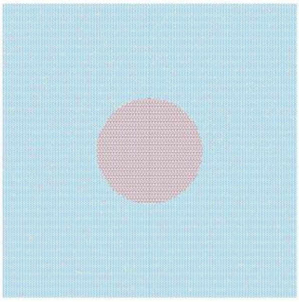	Mg: 771802<br>Al: 25980<br>Ca: 14810	-1311176.28	- 2.89
**6**	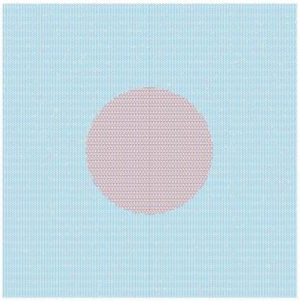	Mg: 771802<br>Al: 25980<br>Ca: 14810	-1313291.98	- 5.50
**7**	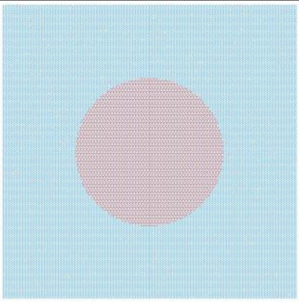	Mg: 771802<br>Al: 25980<br>Ca: 14810	-1314301.07	- 6.74
**8**	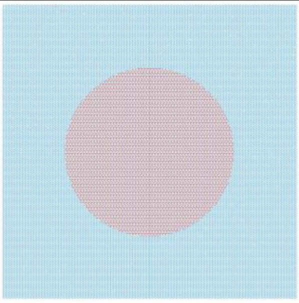	Mg: 771802<br>Al: 25980<br>Ca: 14810	-1315996.70	- 8.82
**9**	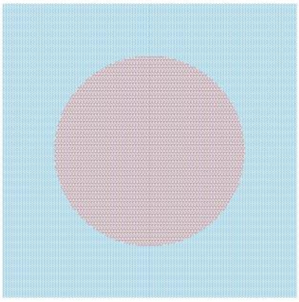	Mg: 771802<br>Al: 25980<br>Ca: 14810	-1321214.81	- 15.24

Supplementary References

[1] Freysoldt, C. et al. First-principles calculations for point defects in solids. *Rev. Mod. Phys*. 86, 253 (2014).

[2] Van de Walle, C. G., & Neugebauer, J. First-principles calculations for defects and impurities: Applications to III-nitrides. *J. Appl. Phys*. 95, 3851 (2004).
