# Supplementary material for: Discovery of confined two-dimensional Laves tiling in a magnesium alloy
Source: Nat Commun. 2026 Apr 15;17:5207. doi: 10.1038/s41467-026-71932-9 (PMC13253817; doi:10.1038/s41467-026-71932-9)
Supplement: Supplementary file 2 — Description of Additional Supplementary Files [file 41467_2026_71932_MOESM2_ESM.pdf]

Description of Additional Supplementary Files

File Name: Supplementary Video 1

Description: Atomic-scale structural optimization of the confined 2D Laves precipitate viewed along the $[0001]_\alpha$ zone axis (corresponding to Fig. 6a). The video demonstrates the transition to the final equilibrium state, revealing the sixfold rotational symmetry and a regular hexagonal arrangement within the basal plane. Blue, yellow, and red spheres represent Mg, Al, and Ca atoms, respectively.

File Name: Supplementary Video 2

Description: Atomic-scale structural optimization of the confined 2D Laves precipitate viewed along the $[\bar{1}100]_\alpha$ zone axis (corresponding to Fig. 6b). The video illustrates the energy minimization and structural relaxation process, showing the resulting densely packed atomic arrangement within the Mg matrix. Blue, yellow, and red spheres represent Mg, Al, and Ca atoms, respectively.

File Name: Supplementary Video 3

Description: Atomic-scale structural optimization of the confined 2D Laves precipitate viewed along the $[11\bar{2}0]_\alpha$ zone axis (corresponding to Fig. 6c). This cross-sectional view highlights the formation of the characteristic structural motif: a rhombic tiling formed by the periodic arrangement of icosahedral clusters (columnar motifs), showing the single unit-cell thickness of the 2D Laves structure. Blue, yellow, and red spheres represent Mg, Al, and Ca atoms, respectively.
