# Supplementary material for: Discovery of confined two-dimensional Laves tiling in a magnesium alloy
Source: Nat Commun. 2026 Apr 15;17:5207. doi: 10.1038/s41467-026-71932-9 (PMC13253817; doi:10.1038/s41467-026-71932-9)
Supplement: Supplementary file 6 — Transparent Peer Review file [file 41467_2026_71932_MOESM6_ESM.pdf]

nature portfolio

Peer Review File

Discovery of confined two-dimensional Laves tiling in a magnesium alloy

Corresponding Author: Professor Hongbo Xie

This file contains all reviewer reports in order by version, followed by all author rebuttals in order by version.

Version 0:

Reviewer comments:

Reviewer #1

(Remarks to the Author)
This work reports the discovery of a confined monolayer-thick Laves structure (with a single-unit-cell thickness) in solute-dilute Mg alloys, which constitutes the fundamental building block of numerous Laves phases. The identification of such a structure is critical for understanding the composition of Laves phases and provides valuable insights into their formation mechanisms and clustering behavior. Furthermore, this study elucidates the aging-hardening mechanism in Mg–Al–Ca alloys by clarifying the precipitate structure and establishing a theoretical foundation for the design of high-strength, high-toughness Mg–Al–Ca alloys. The manuscript is well organized and supported by high-quality experimental data, including atomic-resolution HAADF-STEM images and EDS mapping, as well as comprehensive theoretical analyses (first-principles calculations and molecular dynamics simulations). The results are convincing and, after minor revisions, the work is suitable for publication in Nature Communications.
Specific suggestions for improvement:
1.In the Abstract, the authors state that tensile strain in the matrix suppresses the diffusion of small Al solute atoms and thereby inhibits coarsening of the Laves structure. The underlying mechanism of this effect should be further clarified.
2.The manuscript suggests that this Laves structure is fully coherent with the Mg matrix. Is this truly the case? The degree of lattice misfit in both lateral and longitudinal directions should be evaluated to substantiate this claim.
3.The authors are encouraged to provide a more detailed explanation of the mechanism of interfacial atomic reconstruction during the early growth stage of the Laves structure.
4.The reported thickness of the Laves structure requires clarification—whether it is 4.62 Å or 9.12 Å should be explicitly addressed.

Reviewer #2

(Remarks to the Author)
Xie et al. report on their discovery, and careful characterization, of an isolated Laves tiling within a Mg-2.0wt%Al-1.0wt%Ca alloy that has been aged at 200 degrees Celsius for 24 hours. The experimental findings reported upon within this contribution are undeniably significant and they are well supported by thoughtful computation. Overall, I believe this work is suitable for publication within Nature Communications once my comments and questions below are addressed:

1. The main point of confusion for me is the discussion centered around assigning an Al atom to the center site of their icosahedron. To some degree I think my confusion may arise from the way the atom assignments are initially introduced within the text. My experience with the manuscript is as follows:

As the text is currently structured, I am introduced to the icosahedron motif within Fig. 1. Now, at this point, I have every reason to believe that if I were to observe a Laves tiling with an Al : Ca stoichiometry of 2 : 1, the atom located at the center of the icosahedron should be a Ca atom. However, upon inspection of Fig. 2 (f), it is claimed that Al is located at the center of the experimentally observed icosahedron. Additionally, the authors acknowledge on page 5 of the text that the contrast in HAADF-STEM should scale as Z^{2}, but then insist that the central atom is Al. All of this is very counterintuitive to me. In my opinion, the first sound justification for indexing the central atom as an Al atom appears in Figure 4 (d), where EDS measurements suggest an Al signal at the central site of the icosahedron.

I recommend that the authors rework the discussion centered around indexing the central atom of the icosahedron as an Al atom. Perhaps the simplest way to address this is by not indexing atom types within Fig 2 (f). After all, the main finding of Figure 2 seems to be the observation of icosahedrons arranged in a fashion identical to the laves tiling of Fig. 1 (c). The attempt to also index the atom types in Fig. 2 without first presenting the data in Fig. 3 and Fig. 4 seems distracting.

2. As a follow up, could it be that there is disorder on the central icosahedron site? Perhaps there is both Ca and Al on the central site? Have any first principles calculations been performed to rule out this possibility? Please note that I'm not suggesting that the first principles calculations need to be performed, but I am curious to hear what the authors think.

3. Regarding the molecular dynamics simulations, I have two main questions.

(i) First, the authors state that the precipitate size was "systematically increased to emulate its growth." I would appreciate it if they elaborated on what is meant by this. It looks like they may be defining a "growth step" as a constant change in surface area per effective "time step." If that is the case, how are the authors moving the necessary number of Al/Ca atoms from the matrix to the precipitate phase boundary? Does this process require that the authors assume a certain diffusion mechanism at the atomic scale?

(ii) If I am understanding correctly, the panels of Figure 9 are cross sections orthogonal to the vantage point used in panel (a) of Figure 8. If that's the case, then I presume the precipitate that they have embedded in their simulation cell is cylindrical and I am observing growth of the circular cross section within Figure 9 (a) – (f)? Could the authors please clarify if my interpretation of the precipitate geometry is correct? I would greatly appreciate it if they specify this geometry somewhere in the manuscript – the methods section would be fine.

Some final minor comments:

(A) I would recommend adding atom labels to panel (a) of Fig. 1 – perhaps using the general chemical formula "AB_{2}" (since the authors use this general formula later on in the text). I know this labelling is somewhat implied, but I would argue the redundancy is helpful.

(B) The authors refer to the C14, C15 and C36 Laves polytypes as MgX_{2}, but then introduce their Laves polytype as Al_{2}Ca. Is this intentional? It does seem to align with their assignment of Al to the center of the icosahedron. Perhaps a more fitting chemical formula is Al(Al,Ca)_{2} ? Implying disorder on the X_{2} sites?

As I said above, overall, I greatly enjoyed reviewing this manuscript. The study is thoughtful and compelling and I believe it to be suitable for publication in Nature Communications upon revision.

Reviewer #3

(Remarks to the Author)
This study reports the discovery of confined two-dimensional Al2Ca Laves tiling in a dilute Mg alloy. Detailed STEM characterizations were performed, coupled with a series of theoretical calculations. These experimental results appear sufficiently comprehensive to explain the existence of this specific Laves tiling within the Mg alloy. After carefully reading this work, several questions remain unresolved. Addressing these issues is essential prior to considering its publication. The specific concerns are as follows:

1. The authors emphasize the fabrication of Al2Ca Laves within the Mg alloy. As elucidated in the introduction, over 1400 distinct Laves phases have been reported. What distinguishes this newly discovered phase? What are the distinctive characteristics of this new Laves phase?

2. Given that the Al2Ca Laves phase forms in the Mg alloy, what was the intended purpose of introducing this phase? An increase in Vickers hardness was demonstrated by meticulously controlling annealing time at 200°C. Is enhancing hardness the primary objective of this work? If so, does the introduction of this Laves phase demonstrate significant advantages over other optimization strategies? The manuscript lacks performance comparisons with reported data, particularly concerning different Laves phase structures. In addition, the strengthening mechanism requires a more detailed discussion.

3. Regarding the formation of the Al_2Ca Laves phase within the Mg alloy, the specific role of the Mg matrix needs detailed elucidation. Could Mg atoms contribute to stabilizing the Laves precipitates? As shown in Fig. 3c, the EDS mapping of the Al_2Ca Laves region does not conclusively rule out the presence of Mg due to the weak Mg signal.

4. The authors should evaluate the local strain at the interface between the Al_2Ca Laves phase and the surrounding Mg alloy host. For instance, Geometric Phase Analysis (GPA) calculations should be performed.

5. Is the size distribution of the precipitates influenced by annealing time, and does this subsequently affect the final hardness? Quantification of size evolution is lacking; only image data for 24 h and 500h are provided.

In summary, the work primarily reports the discovery of a novel type of Al3Ca Laves phase but fails to provide a clear rationale for the main research objective. Although extensive first-principles calculations and MD simulations are presented, the construction and detailed analysis of the local coherent interface between the Al_2Ca Laves and the Mg matrix need careful consideration. This is particularly crucial for the interfacial transition region, as local strain critically impacts formation energy. Consequently, I cannot recommend the manuscript in its current version.

Version 1:

Reviewer comments:

Reviewer #1

(Remarks to the Author)
The authors have addressed all my concerns. I would like to recommend this manuscript to be published in Nature Communications.

Reviewer #2

(Remarks to the Author)
I would like to thank the authors for crafting a thoughtful, and detailed, response letter. They have adequately addressed all of my comments/concerns.

This manuscript is now suitable for publication in Nature Communications.

Reviewer #3

(Remarks to the Author)
Thank you for the authors' detailed response. In the revised manuscript, the authors have supplemented the STEM experimental characterization and associated theoretical calculations. These additions strongly support the claim regarding the importance of the newly identified Al_2Ca Laves phase. All of my concerns have been addressed satisfactorily, and I therefore recommend publication in Nature Communications.
Minor suggestion:
I suggest that the authors put the discussion of the Mg element in the EDS characterization of the Al_2Ca phase region presented in Fig. R8 in the main text. This would provide a more comprehensive understanding of the elemental analysis in this area.

Open Access This Peer Review File is licensed under a Creative Commons Attribution 4.0 International License, which permits use, sharing, adaptation, distribution and reproduction in any medium or format, as long as you give appropriate credit to the original author(s) and the source, provide a link to the Creative Commons license, and indicate if changes were made.
In cases where reviewers are anonymous, credit should be given to 'Anonymous Referee' and the source.
The images or other third party material in this Peer Review File are included in the article's Creative Commons license, unless indicated otherwise in a credit line to the material. If material is not included in the article's Creative Commons license and your intended use is not permitted by statutory regulation or exceeds the permitted use, you will need to obtain permission directly from the copyright holder.
To view a copy of this license, visit https://creativecommons.org/licenses/by/4.0/

Point-by-point response to the reviewers' comments

Reviewer #1:

This work reports the discovery of a confined monolayer-thick Laves structure (with a single-unit-cell thickness) in solute-dilute Mg alloys, which constitutes the fundamental building block of numerous Laves phases. The identification of such a structure is critical for understanding the composition of Laves phases and provides valuable insights into their formation mechanisms and clustering behavior. Furthermore, this study elucidates the aging-hardening mechanism in Mg-Al-Ca alloys by clarifying the precipitate structure and establishing a theoretical foundation for the design of high-strength, high-toughness Mg-Al-Ca alloys. The manuscript is well organized and supported by high-quality experimental data, including atomic-resolution HAADF-STEM images and EDS mapping, as well as comprehensive theoretical analyses (first-principles calculations and molecular dynamics simulations). The results are convincing and, after minor revisions, the work is suitable for publication in Nature Communications.

Response:

Thanks for your positive and professional comments on our work. We are very grateful to you for the constructive criticism, which has helped us improve the quality of the manuscript.

Specific suggestions for improvement:

1. In the Abstract, the authors state that tensile strain in the matrix suppresses the diffusion of small Al solute atoms and thereby inhibits coarsening of the Laves structure. The underlying mechanism of this effect should be further clarified.

Response:

We thank the reviewer for this valuable and insightful comment. To elucidate the atomistic mechanism underlying the observed behavior, we performed additional calculations of the segregation energy (ΔE) for an Al solute at a series of lattice sites surrounding the confined Al_2Ca Laves precipitate, using a site in the bulk Mg matrix far from the precipitate as the reference state.

These calculations reveal a highly anisotropic energy landscape governed by the local strain field (Supplementary Figure R1). Specifically:

1. Sites located directly above the precipitate on the Mg (0001) basal plane experience a pronounced tensile strain field and exhibit positive ΔE values. This introduces a substantial

energetic penalty, rendering these positions unfavourable for Al solutes and thereby impeding direct diffusion and attachment to the top interface.

2. Sites along the lateral sides of the precipitate display negative ΔE values, indicating that these regions are energetically favourable for Al segregation and facilitate solute diffusion along the precipitate flanks.

This spatially heterogeneous energy landscape provides a mechanistic basis for the inhibition of coarsening. The tensile strain field does not completely obstruct Al transport; rather, it introduces a kinetic bottleneck by selectively suppressing atomic attachment to the broad, coherent interface—the most efficient pathway for lateral growth of the Laves structure. As a result, precipitate growth is effectively restrained, consistent with the experimentally observed suppression of coarsening.

We have incorporated this discussion, together with the corresponding figure, into the revised manuscript. We thank the reviewer again for prompting this deeper mechanistic clarification.

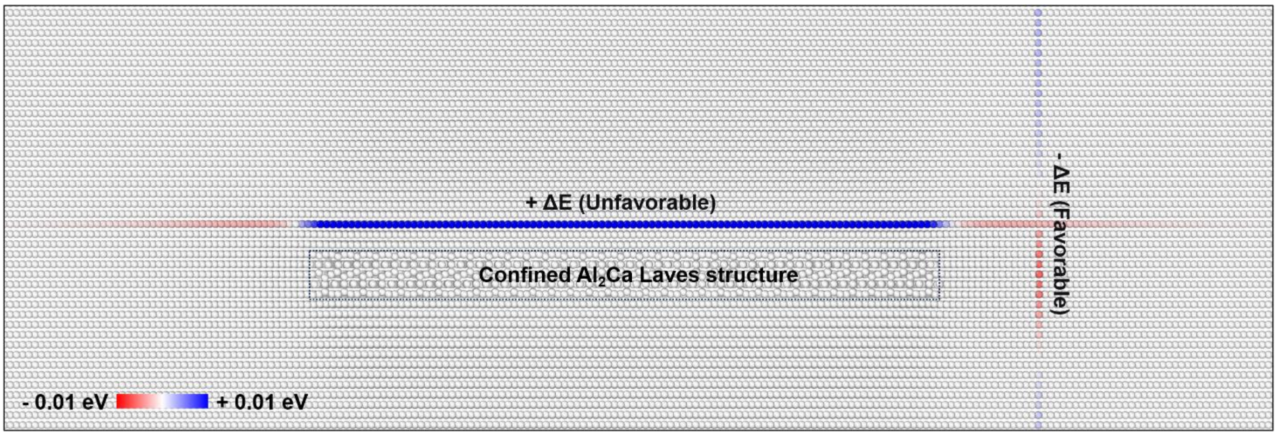

Figure R1. Schematic illustration of the asymmetric energy landscape for Al-solute diffusion near the confined 2D Laves precipitate. The tensile strain field above the precipitate presents an energy barrier, whereas the lateral regions provide energetically favourable sites that facilitate solute diffusion.

2. The manuscript suggests that this Laves structure is fully coherent with the Mg matrix. Is this truly the case? The degree of lattice misfit in both lateral and longitudinal directions should be evaluated to substantiate this claim.

Response:

We thank the reviewer for this important suggestion. To quantitatively substantiate the

coherency of the interface, we performed first-principles calculations to determine the lattice parameters of both the confined 2D Laves precipitate and the Mg matrix, subsequently evaluating the interfacial misfit.

The results clearly support our assertion of a fully coherent interface:

Lateral misfit (basal plane):

$[1\bar{1}00]\alpha$ direction: 3.00%

$[11\bar{2}0]\alpha$ direction: 2.99%

Longitudinal misfit (growth direction): 10.45%

The remarkably small lateral misfits (≈3%) lie well below the conventional threshold for coherency (<5%), indicating that the in-plane mismatch can be readily accommodated without the formation of misfit dislocations. This minimal lateral strain is the key factor enabling the Laves phase to form a fully coherent interface with the Mg matrix.

Although the longitudinal misfit is larger, it is accommodated elastically, as evidenced by the uniform atomic registry and the absence of interfacial dislocations in our high-resolution image (Figure 2b). Such behavior is characteristic of heteroepitaxial interfaces that remain coherent while sustaining elastic strain.

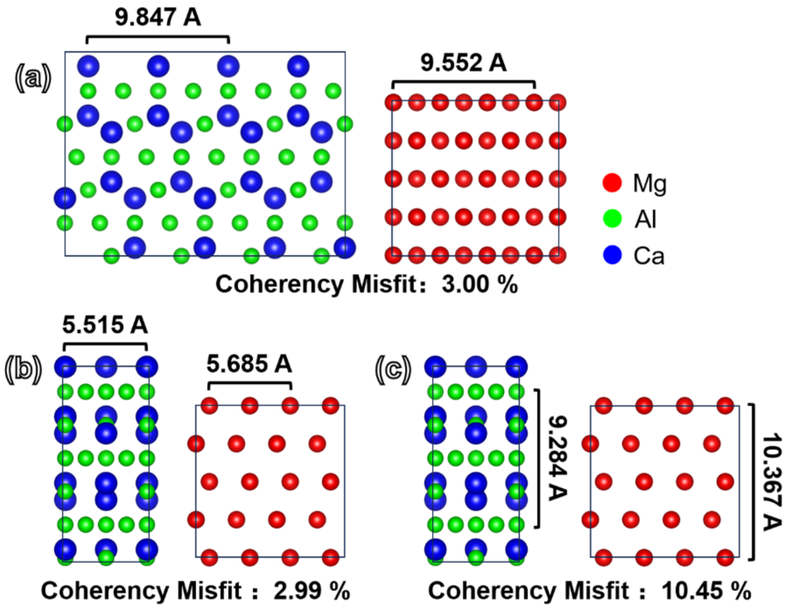

Figure R2. The coherency misfit along the three directions between the Al_2Ca Laves phase and the Mg matrix, calculated from first-principles derived lattice parameters.

3. The authors are encouraged to provide a more detailed explanation of the mechanism of interfacial atomic reconstruction during the early growth stage of the Laves structure.

Response:

We thank the reviewer for this valuable suggestion. To elucidate the atomistic mechanism underlying the interfacial reconstruction, we performed *ab initio* structural-relaxation calculations to simulate the initial formation of the confined 2D Laves structure from a co-segregated solute layer within the Mg matrix.

These calculations reveal that the reconstruction proceeds as a barrier-less, spontaneous, and cooperative process driven by the thermodynamic preference for forming the topologically close-packed Laves structure. We constructed an initial atomic model in which Al and Ca atoms occupy specific HCP lattice sites on the Mg basal plane, and evaluated the transformation from three principal crystallographic orientations to confirm its robustness.

Strikingly, in all cases the initially disordered solute layer spontaneously reconstructs into the characteristic monolayer Al_2Ca Laves phase. The accompanying simulations (Supplementary Videos S1–S3) vividly capture the transformation pathway, which comprises two key steps:

Cooperative in-plane shear: The solute raft undergoes a collective shear distortion within the basal plane, providing the structural pathway necessary to achieve the new atomic packing.

Formation of the Laves framework: This shear motion drives the Al and Ca sublattices into the characteristic Laves configuration, establishing a coherent interface with the Mg matrix. In the final arrangement, Al atoms occupy the centres of the emerging icosahedral clusters.

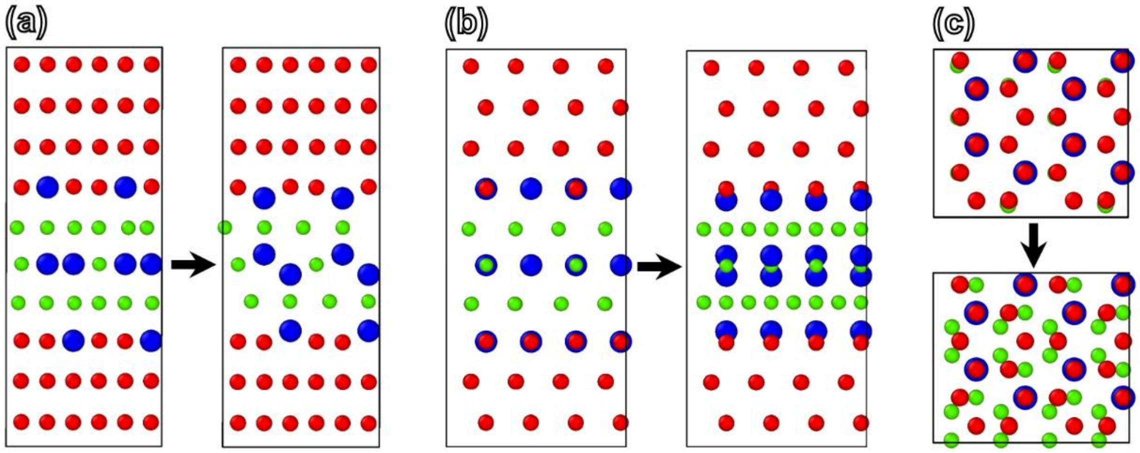

Figure R3. Atomistic mechanism of the spontaneous interfacial reconstruction leading to the formation of the confined 2D Laves structure.

Together, these results demonstrate that the formation of the 2D Laves phase is both thermodynamically favourable and kinetically accessible, arising naturally from a solute-rich

precursor layer. This mechanism is summarized in a new schematic (Supplementary Fig. R3), and the associated videos have been cited in the revised manuscript.

4. The reported thickness of the Laves structure requires clarification—whether it is 4.62 Å or 9.12 Å should be explicitly addressed.

Response:

We thank the reviewer for requesting this important clarification. The value of 9.12 Å corresponds to the total thickness of the experimentally observed confined 2D Laves structure.

The two values are hierarchically related:

- **4.62 Å: This is the height of the fundamental structural building block of the Laves phase.**
- **9.12 Å: This is the full thickness of the confined monolayer.**

The revised manuscript now explicitly distinguishes the total precipitate thickness from the subunit height to eliminate any ambiguity.

Reviewer #2:

Xie et al. report on their discovery, and careful characterization, of an isolated Laves tiling within a Mg-2.0wt%Al-1.0wt%Ca alloy that has been aged at 200 degrees Celsius for 24 hours. The experimental findings reported upon within this contribution are undeniably significant and they are well supported by thoughtful computation. Overall, I believe this work is suitable for publication within Nature Communications once my comments and questions below are addressed:

Response:

Thanks for your positive and professional comments on our work. We are very grateful to you for the constructive criticism which has helped us to improve the quality of the manuscript.

1. The main point of confusion for me is the discussion centered around assigning an Al atom to the center site of their icosahedron. To some degree I think my confusion may arise from the way the atom assignments are initially introduced within the text. My experience with the manuscript is as follows:

As the text is currently structured, I am introduced to the icosahedron motif within Fig. 1. Now, at this point, I have every reason to believe that if I were to observe a Laves tiling with an Al: Ca stoichiometry of 2: 1, the atom located at the center of the icosahedron should be a Ca atom. However,

upon inspection of Fig. 2 (f), it is claimed that Al is located at the center of the experimentally observed icosahedron. Additionally, the authors acknowledge on page 5 of the text that the contrast in HAADF-STEM should scale as Z^ {2}, but then insist that the central atom is Al. All of this is very counterintuitive to me. In my opinion, the first sound justification for indexing the central atom as an Al atom appears in Figure 4 (d), where EDS measurements suggest an Al signal at the central site of the icosahedron.

I recommend that the authors rework the discussion centered around indexing the central atom of the icosahedron as an Al atom. Perhaps the simplest way to address this is by not indexing atom types within Fig 2 (f). After all, the main finding of Figure 2 seems to be the observation of icosahedrons arranged in a fashion identical to the laves tiling of Fig. 1 (c). The attempt to also index the atom types in Fig. 2 without first presenting the data in Fig. 3 and Fig. 4 seems distracting.

Response:

We thank the reviewer for this insightful comment and excellent suggestion regarding the presentation of Figure 2. We agree that introducing atomic indexing before presenting the chemical evidence (EDS) could create confusion. Following the reviewer's advice, we have removed the atomic labels (Al/Ca) from Fig. 2(f) in the revised manuscript. The figure now focuses solely on demonstrating the structural match between the observed atomic arrangement and the Laves phase tiling model, thereby avoiding premature chemical assignment.

To comprehensively address the reviewer's valid concern regarding the counterintuitive assignment of Al (a lighter element) to the central site, we have conducted a multi-faceted analysis. We present three independent lines of evidence confirming that Al occupies the central position:

1. Quantitative HAADF-STEM Contrast Analysis & Image Simulation

The reviewer correctly notes that HAADF contrast generally scales with Z^2. However, the projected intensity also depends linearly on the number of atoms within the atomic column.

Atomic Density Compensation: As illustrated in Supplementary Figure R4 below, the central column of the icosahedron consists of four Al atoms per unit cell height, whereas the surrounding network columns consist of only two Ca atoms.

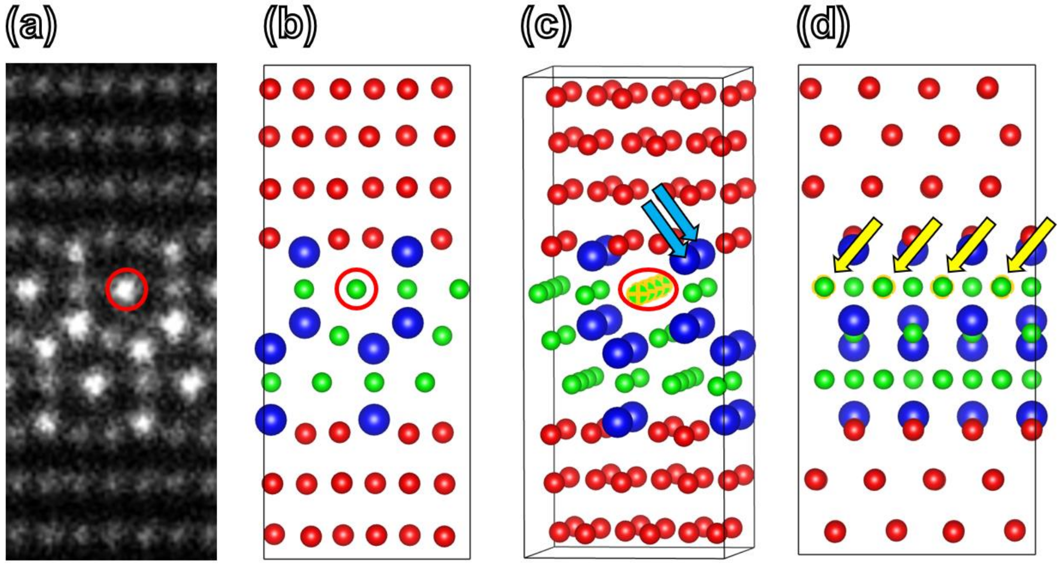

Figure R4. Schematic of the atomic column composition within the confined 2D Laves structure. The similar HAADF-STEM intensity observed at the central and network sites results from the higher atomic count (4 Al) in the central column, which compensates for its lower atomic number relative to the network columns (2 Ca).

Image Simulation: The combined scattering power of four Al atoms results in an intensity comparable to that of two Ca atoms. To validate this, we performed multislice HAADF image simulations, which are now included as Supplementary Figure S3 in the revised manuscript. The simulations perfectly reproduce the experimental observation where the central (Al) and network (Ca) sites exhibit similar brightness, confirming that the contrast is modulated by column atomic density.

2. First-Principles Energetics: A Comprehensive Assessment

To definitively rule out the possibility of Ca occupying the central site, we calculated the formation energies for various substitutional defect configurations (Supplementary Figure R5).

● Configurations Tested: We systematically modeled Ca substituting for Al at the central site in multiple patterns: (a) single-atom substitution, (b–e) two-atom substitutions in different spatial arrangements, and (f) full substitution of the central column (4 Ca atoms).

● Unambiguous Result: All calculated configurations exhibit significantly positive defect formation energies. This indicates that placing Ca at the center is thermodynamically highly unfavorable, regardless of the specific local arrangement or clustering.

Calculation Methodology: To systematically evaluate the thermodynamic stability of various site occupations, we calculated the substitutional defect formation energy E_f using the

general formalism [1, 2]:

$$E_f = E_{defect} - E_{ref} - n(\mu_M - \mu_H)$$

where:

E_{defect} is the total energy of the supercell containing the substitutional defect.

E_{ref} is the total energy of the pristine, reference supercell.

n is the number of atoms being substituted.

μ_M is the chemical potential of the substituting element (the dopant/solute entering the lattice).

μ_H is the chemical potential of the host element (the atom being replaced at the specific lattice site).

In the specific context of the reviewer's question (Ca occupying the central site), M corresponds to Ca and H corresponds to Al.

A positive value of E_f indicates that the substitution is thermodynamically unfavorable.

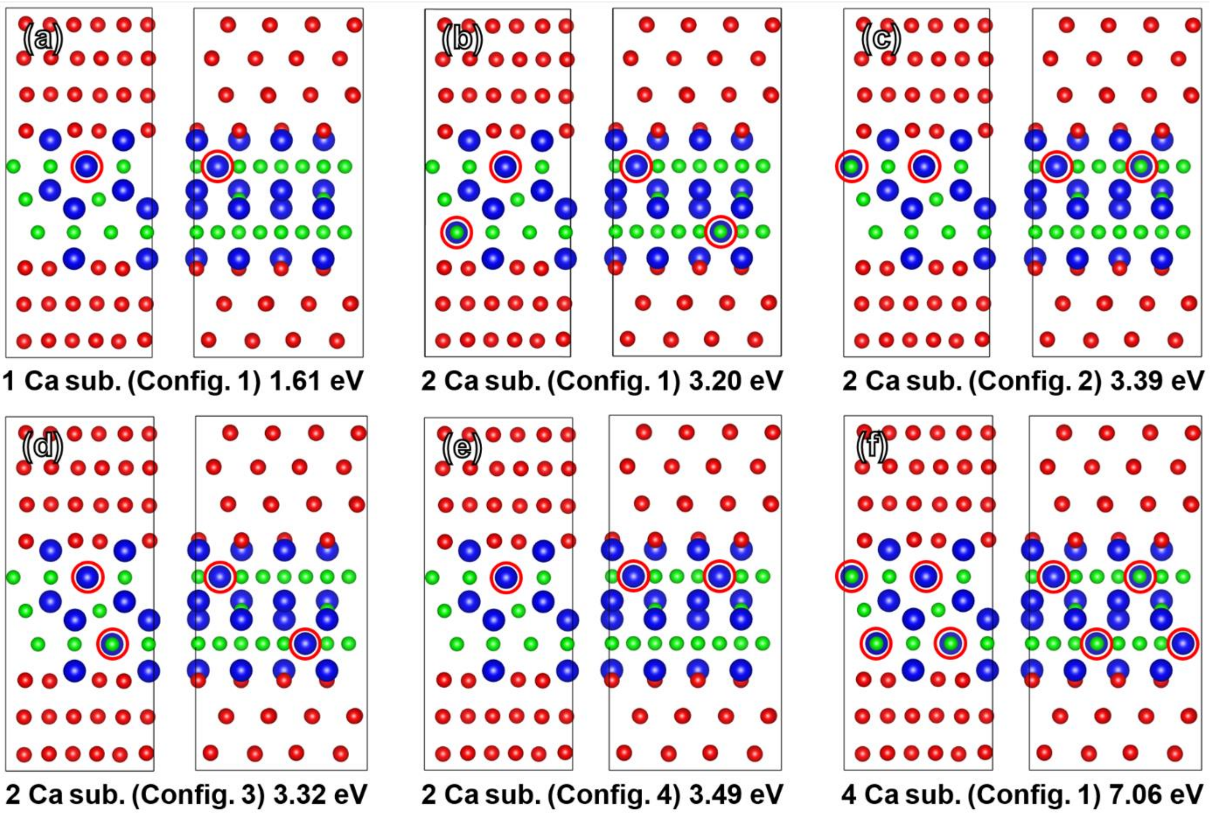

Figure R5. Energetic instability of Ca substitutions at the central icosahedral site. **(a-f)** Atomic structures of the six calculated defect configurations, where Al atoms at the central site are substituted by Ca atoms in different patterns. The corresponding substitutional defect formation energy (in eV)

is indicated for each configuration. The large positive values in all cases conclusively demonstrate that placing Ca at the center is thermodynamically highly unfavorable.

3. Dynamic Formation Mechanism via DFT Structural Relaxation:

Our *ab initio* structural relaxation calculations (visualized in Supplementary Videos S1–S3) capture the barrier-less, spontaneous reconstruction of the Laves structure from the initial solute layer. The evolution of atomic coordinates unambiguously shows that Al atoms naturally and consistently migrate to occupy the centers of the emerging icosahedral clusters. This provides direct evidence that the central site is the inherent, preferred position for Al as the system relaxes to its energy minimum.

We believe this rigorous combination of image simulation, thermodynamic calculations, and spontaneous reconstruction trajectories thoroughly resolves the counterintuitive contrast and solidifies our atomic assignment.

References

[1] Freysoldt, C. et al. First-principles calculations for point defects in solids. *Rev. Mod. Phys.* **86**, 253 (2014).

[2] Van de Walle, C. G., & Neugebauer, J. First-principles calculations for defects and impurities: Applications to III-nitrides. *J. Appl. Phys.* **95**, 3851 (2004).

2. As a follow up, could it be that there is disorder on the central icosahedron site? Perhaps there is both Ca and Al on the central site? Have any first principles calculations been performed to rule out this possibility? Please note that I'm not suggesting that the first principles calculations need to be performed, but I am curious to hear what the authors think.

Response:

We thank the reviewer for this insightful question. We have indeed performed first-principles calculations to explicitly investigate this possibility, and our results strongly indicate that the central site maintains a high degree of Al ordering.

Our comprehensive substitutional defect formation energy calculations (presented in Figure R5 of this letter) tested six different configurations where Ca atoms replace Al at the central site. These ranged from single atom substitutions to full column replacements. The results lead to the following conclusions: This high energy cost leads us to two key conclusions:

1. Strong Energetic Preference for Ordering: Our calculations reveal that substituting Ca

into the central Al site is energetically expensive. All tested configurations resulted in significantly positive defect formation energies, with the lowest energy penalty being 1.608 eV per substituted atom.

Thermodynamic Implication: This substantial energy cost (>1.6 eV) implies a powerful thermodynamic driving force for Al to exclusively occupy the central site. At the experimental aging temperature of 200°C, the Boltzmann probability of thermally activating such antisite defects is negligible. Therefore, the equilibrium structure is predicted to be highly ordered.

2. Physical Origin of the Order (Strain & Size Mismatch): We agree with the reviewer that absolute perfection is rare in materials. However, from a physical perspective, the significant atomic size mismatch between Ca (r_{Ca}≈1.97 Å) and Al (r_{Al}≈1.43 Å) makes the central icosahedral site sterically unfavorable for Ca. Placing the much larger Ca atom in the confined central site would induce severe local strain, thereby destabilizing the precise topology of the Laves structure.

3. Regarding the molecular dynamics simulations, I have two main questions.

(i) First, the authors state that the precipitate size was "systematically increased to emulate its growth." I would appreciate it if they elaborated on what is meant by this. It looks like they may be defining a "growth step" as a constant change in surface area per effective "time step." If that is the case, how are the authors moving the necessary number of Al/Ca atoms from the matrix to the precipitate phase boundary? Does this process require that the authors assume a certain diffusion mechanism at the atomic scale?

Response:

We thank the reviewer for this excellent question, which allows us to clarify the nature and objective of our molecular dynamics (MD) simulations. The reviewer's intuition is correct; our approach is best described as a non-equilibrium "virtual" growth experiment rather than a simulation of the actual diffusion-controlled growth process.

We elaborate on the procedure as follows:

(a) Initial Configuration: We started with an atomic model containing a small, coherent Laves phase precipitate embedded within an Mg matrix supersaturated with Al and Ca solutes.

(b) "Growth Step" Protocol: The precipitate was enlarged not by natural diffusion over time, but by manually and iteratively swapping Mg atoms at the precipitate/matrix interface

with nearby solute (Al/Ca) atoms in the matrix. This artificially enriches the interface with the necessary precipitate-forming elements.

(c) Relaxation: After each atomic swap, the entire system was subjected to energy minimization and subsequent MD relaxation at the target temperature to allow the local atomic structure and strain field to equilibrate into a (meta)stable state.

The physical interpretation of this method is that it simulates growth in the limit of infinitely fast diffusion. It directly imposes the chemical driving force for precipitation while bypassing the kinetic limitations of atomic migration. This allows us to isolate and answer a specific question: Under a strong thermodynamic driving force, how does the interface structure, coherency, and strain field evolve as the precipitate grows?

We explicitly acknowledge the limitation that this method does not simulate the specific atomic-scale diffusion mechanism (e.g., vacancy-mediated jumps). The simulation timescale of our MD, while sufficient for local structural relaxation (picoseconds to nanoseconds), is far too short to capture the real diffusion kinetics that occur over microseconds to seconds. Therefore, our simulation is a study of thermodynamically driven interface evolution, not a reproduction of the kinetics of the growth process.

We have revised the Methods section to include this detailed explanation and to clearly state the scope and limitations of this particular simulation.

(ii) If I am understanding correctly, the panels of Figure 9 are cross sections orthogonal to the vantage point used in panel (a) of Figure 8. If that's the case, then I presume the precipitate that they have embedded in their simulation cell is cylindrical and I am observing growth of the circular cross section within Figure 9 (a) - (f)? Could the authors please clarify if my interpretation of the precipitate geometry is correct? I would greatly appreciate it if they specify this geometry somewhere in the manuscript - the methods section would be fine.

Response:

We thank the reviewer for the comment, and we confirm that your understanding is entirely correct. The three-dimensional atom probe reconstruction (Figure R6) provides the most direct visualization of the precipitate morphology, revealing that the confined 2D Laves structure adopts an approximately circular, disk-like geometry with a diameter of ~40 nm.

Guided by this experimentally derived morphology, the precipitate in the simulation cell

was modelled as a disk-shaped cylinder. Accordingly, the panels in Figure 9 present cross-sections taken perpendicular to the viewing direction in Fig. 8a, thereby illustrating the radial growth of the precipitate's circular cross-section.

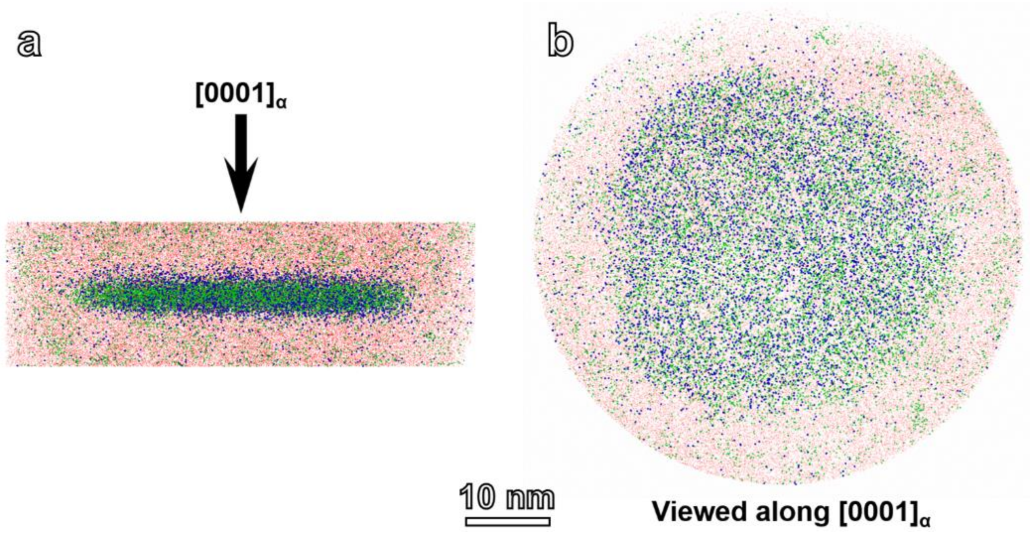

Figure R6. APT reveals the morphology of the confined 2D Laves precipitate within the Mg matrix.

Some final minor comments:

(A) I would recommend adding atom labels to panel (a) of Fig. 1 - perhaps using the general chemical formula "AB_{2}" (since the authors use this general formula later on in the text). I know this labelling is somewhat implied, but I would argue the redundancy is helpful.

Response:

We sincerely thank the reviewer for this constructive suggestion. We agree that adding atom labels to panel (a) of Fig. 1 would enhance clarity, particularly as the "AB_2" general formula is later introduced in the text. To address this, we have now labeled the panel with "AB_2" to explicitly connect the atomic motif to the broader Laves phase context, while maintaining the visual simplicity of the original figure. This redundancy, as the reviewer rightly noted, will help readers quickly associate the observed atomic arrangement with the Laves structure terminology used throughout the manuscript.

(B) The authors refer to the C14, C15 and C36 Laves polytypes as MgX_ {2}, but then introduce their Laves polytype as Al_ {2} Ca. Is this intentional? It does seem to align with their assignment of Al to the center of the icosahedron. Perhaps a more fitting chemical formula is Al(Al,Ca)_ {2}? Implying disorder on the X_ {2} sites?

Response:

We thank the reviewer for this insightful comment. Laves phases are topologically close-packed AB_2 structures comprising large (A) and small (B) constituent atoms. The C14, C15, and C36 prototypes correspond to $MgZn_2$, $MgCu_2$, and $MgNi_2$, respectively, in which Mg (160 pm) occupies the larger A sites, whereas Zn (134 pm), Cu (128 pm), and Ni (124 pm) occupy the smaller B sites. In the Al_2Ca structure, by contrast, Ca (197 pm) serves as the larger A atom and Al (143 pm) as the smaller B atom.

Consistent with our first-principles calculations presented above, substituting Ca into the icosahedral-centre Al site leads to a substantial destabilisation of the lattice. This conclusion is further supported by EDS mapping (Figure 4), which clearly shows that the centres of the icosahedral columns are occupied by Al. Therefore, the structure is identified as Al_2Ca.

As I said above, overall, I greatly enjoyed reviewing this manuscript. The study is thoughtful and compelling and I believe it to be suitable for publication in Nature Communications upon revision.

Response:

We sincerely thank the reviewer for their positive assessment of the significance of our study and the constructive nature of their comments.

Reviewer #3:

This study reports the discovery of confined two-dimensional Al2Ca Laves tiling in a dilute Mg alloy. Detailed STEM characterizations were performed, coupled with a series of theoretical calculations. These experimental results appear sufficiently comprehensive to explain the existence of this specific Laves tiling within the Mg alloy. After carefully reading this work, several questions remain unresolved. Addressing these issues is essential prior to considering its publication. The specific concerns are as follows:

Response:

Thank you for your professional feedback on our work. We sincerely appreciate your constructive criticism, which has greatly contributed to the improvement of the manuscript quality. We now proceed systematically to address each of the concerns you have raised, individually and in a methodical manner.

1. The authors emphasize the fabrication of Al2Ca Laves within the Mg alloy. As elucidated in the introduction, over 1400 distinct Laves phases have been reported. What distinguishes this newly discovered phase? What are the distinctive characteristics of this new Laves phase?

Response:

We appreciate the reviewer's thoughtful comments. Among the more than 1400 reported Laves phases—most of which adopt the canonical C14 ($MgZn_2$), C15 ($MgCu_2$), or C36 ($MgNi_2$) prototypes—the underlying structural motif can be traced to a common 2D Laves building block (Figure 1 in manuscript). With the advent of aberration-corrected STEM, a far broader structural landscape has come into view, including aperiodic arrangements and even quasicrystalline configurations with fivefold rotational symmetry, all of which are likewise assembled from this 2D Laves building block. Recognizing this elementary 2D Laves building block is therefore essential for understanding the structural diversity and topological complexity of Laves-type compounds.

In this study, the 2D Laves motif identified in the Mg–Al–Ca system represents a fundamental structural unit from which numerous Laves variants may arise. In this work, we resolve—at atomic resolution—how this confined 2D Laves building block is intricately embedded within the Mg matrix, and we elucidate, through theory, the nucleation pathway, growth mechanism, and physical origin of its resistance to coarsening.

Collectively, these findings reveal a structurally confined Laves-tiling motif and provide key insight into the nucleation, stabilization, and transformation of Laves phases, thereby establishing a bridge between classical bulk Laves structures and their nanoscale counterparts.

2. Given that the Al2Ca Laves phase forms in the Mg alloy, what was the intended purpose of introducing this phase? An increase in Vickers hardness was demonstrated by meticulously controlling annealing time at 200°C. Is enhancing hardness the primary objective of this work? If so, does the introduction of this Laves phase demonstrate significant advantages over other optimization strategies? The manuscript lacks performance comparisons with reported data, particularly concerning different Laves phase structures. In addition, the strengthening mechanism requires a more detailed discussion.

Response:

We sincerely thank the reviewer for raising these important points regarding the purpose

and mechanical implications of the Al_2Ca Laves phase in our study. We appreciate the opportunity to clarify the scope and contributions of our work.

The central objective of this study is the discovery and atomic-scale characterization of a previously unreported confined monolayer-thick Laves structure in a solute-dilute Mg-Al-Ca alloy. As highlighted by Reviewers #1 and #2, the identification of such a fundamental Laves building block is of broad significance for understanding the composition-structure relationship and formation mechanisms of Laves phases in general. While mechanical performance was not the primary focus, we included hardness measurements and a discussion of strengthening mechanisms to demonstrate that the identified nanostructure has a tangible macroscopic effect and exhibits potential as a strengthening phase.

That said, we fully agree with the reviewer that a deeper contextualization of the mechanical behavior is valuable. In response, we have now included a more detailed discussion of the strengthening mechanisms in the revised manuscript. Specifically, we have:

Added references to key literature that establish the favorable deformation behavior of Al_2Ca compared to more brittle Laves phases [1, 2], underscoring why this phase is a promising candidate for achieving a balance between strength and ductility.

It is worth emphasizing that the Al_2Ca phase was selected not merely for hardness enhancement, but due to its recognized potential for combining strengthening with retained deformability - a rarity among Laves phases. The exceptional thermal stability of Al_2Ca, as documented in studies on creep-resistant Mg alloys [3], further justifies its investigation. The confined, coherent nature of the monolayer precipitate identified here offers a new microstructural design pathway to leverage these benefits.

In summary, while the core of this paper remains the discovery and atomic-level elucidation of a new form of Laves phase, we have strengthened the mechanical property discussion in the revised version to better address its implications and situate our findings within the existing field. We are confident that these revisions provide a more complete picture and clearly outline the scientific and practical significance of the discovered structure.

References

[1] Zhu, G., et al. Highly deformable Mg-Al-Ca alloy with Al_2Ca precipitates. *Acta Mater*. **200,** 236-245 (2020).

[2] Freund, M., et al. Influence of chemical composition on the room temperature plasticity of C15

Ca-Al-Mg Laves phases. *Acta Mater.* **276,** 120124 (2024).

[3] Cui, Z., et al. Enhancing the creep resistance in a RE-free cast Mg-Al-Ca alloy through microalloying of Ti. *J. Magnes. Alloys* **13,** 414-428 (2025).

3. Regarding the formation of the Al_2Ca Laves phase within the Mg alloy, the specific role of the Mg matrix needs detailed elucidation. Could Mg atoms contribute to stabilizing the Laves precipitates? As shown in Fig. 3c, the EDS mapping of the Al_2Ca Laves region does not conclusively rule out the presence of Mg due to the weak Mg signal.

Response:

We thank the reviewer for this critical question regarding the role of the Mg matrix. The stabilization provided by the Mg matrix is indeed fundamental but operates indirectly through the interface, rather than through Mg solubility within the precipitate itself. Our combined experimental and computational analysis leads to the following conclusions:

1. The Mg Matrix Acts as a Stabilizing "Template" via a Low-Energy Interface:

The primary role of the Mg matrix is not to supply Mg atoms to the precipitate, but to provide a crystalline template that allows the Al_2Ca phase to form a low-energy, coherent interface. The observed clear orientation relationship indicates excellent atomic matching at the interface. This coherent bonding dramatically lowers the interfacial energy, which is a critical factor for both the nucleation and stability of the precipitate. In essence, the Mg matrix stabilizes the Laves phase by accommodating it with minimal structural penalty at the boundary.

2. First-Principles Calculations Rule Out Significant Mg Solubility in the Precipitate:

To directly address whether Mg atoms can incorporate into the Laves phase, we systematically calculated the substitutional defect formation energy. Crucially, to ensure our conclusions were robust against finite-size effects and relevant across a range of concentrations, we performed these calculations using two different supercell sizes (1×1×1 and a larger 1×2×1 model). This approach allowed us to probe Mg substitutions at effective concentrations ranging from 12.5% to 50%, depending on the specific lattice site. The calculation methodology and corresponding atomic sites are illustrated in Figure R7, while the detailed formation energy results across all configurations are summarized in Table R1.

The results are unequivocal: across all five distinct lattice sites and both supercell sizes,

every calculated formation energy is positive, with values ranging from ~0.5 eV to ~0.9 eV. The lowest energy we found was 0.499 eV, which represents a substantial energy penalty. At the experimental aging temperature of 200°C, the thermodynamic probability for such a substitution is vanishingly low. This consistent positivity across different configurations and concentrations definitively demonstrates that Mg incorporation is thermodynamically prohibited. It confirms that the Al_2Ca Laves phase is an inherently stable compound, and the introduction of Mg (with its different atomic size) would destabilize its precise topological close-packing.

3. The Weak Mg EDS Signal is Attributable to Signal Overlap from the Mg Matrix:

Given the computational evidence above, the most plausible explanation for the weak Mg signal is signal overlap from the surrounding Mg matrix—a common technical artifact when analyzing embedded nanoscale precipitates via EDS. This arises because the electron beam's interaction volume often exceeds the precipitate's dimensions, inevitably exciting X-rays from the matrix material surrounding or underlying the precipitate. Crucially, this is further corroborated by the atomic-resolution analysis in Figure R8: the lattice sites corresponding to the Mg signal do not align with the intrinsic structure of the Laves phase. Instead, they retain the characteristic ABAB stacking sequence of the α-Mg matrix. This structural continuity provides direct evidence that the detected Mg signal originates from the matrix background (i.e., the "matrix effect") rather than indicating genuine Mg solubility within the precipitate

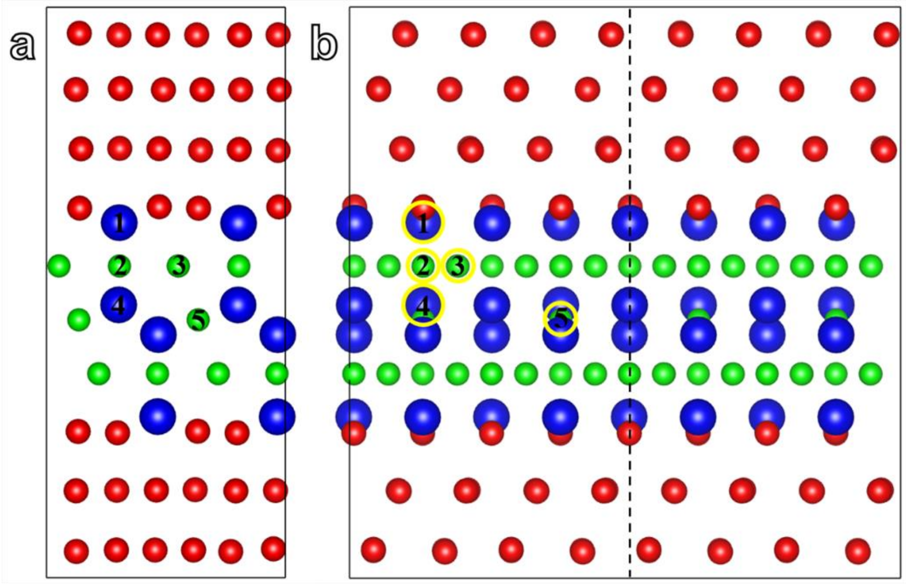

Figure R7. Atomic models used to evaluate the thermodynamic stability of Mg atom substitution in the confined 2D Laves precipitate.

Table R1. Calculated formation energies of substitutional defects for Mg atoms at different lattice sites, demonstrating thermodynamic unfavorability.

	1	2	3	4	5
1*1*1	0.815 eV	0.509 eV	0.499 eV	0.912 eV	0.586 eV
1*2*1	0.780 eV	0.522 eV	0.633 eV	0.869 eV	0.593 eV

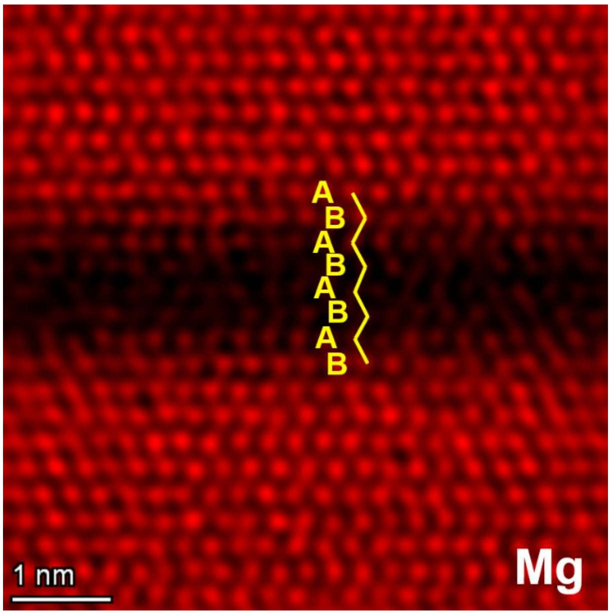

Figure R8. EDS mapping of the confined 2D Laves precipitate within the Mg matrix, showing that the Mg signal originates from lattice sites inconsistent with the Laves crystal structure, and instead retains the characteristic ABAB stacking sequence of the α-Mg matrix.

In summary, the Mg matrix plays a crucial indirect role by stabilizing the Laves precipitate through the formation of a coherent interface. However, direct stabilization via Mg solubility in the Al_2Ca phase is thermodynamically highly unfavorable, as proven by our first-principles calculations. Therefore, we are confident that the precipitate is predominantly stoichiometric Al_2Ca, with the observed Mg signal originating from the matrix. We have enriched the discussion in the manuscript with this multi-faceted analysis to provide a detailed elucidation of the Mg matrix's role.

Calculation Methodology: To systematically evaluate the thermodynamic stability of various site occupations, we calculated the substitutional defect formation energy E_f using the general formalism [1, 2]:

$$E_f = E_{defect} - E_{ref} - n(\mu_M - \mu_H)$$

where:

E_{defect} is the total energy of the supercell containing the substitutional defect.

E_{ref} is the total energy of the pristine, reference supercell.

***n* is the number of atoms being substituted.**

μ_M is the chemical potential of the substituting element (the dopant/solute entering the lattice).

μ_H is the chemical potential of the host element (the atom being replaced at the specific lattice site).

In the specific context of the reviewer's question, M corresponds to Mg and H corresponds to Ca/Al.

A positive value of E_f indicates that the substitution is thermodynamically unfavorable.

References

[1] Freysoldt, C. et al. First-principles calculations for point defects in solids. *Rev. Mod. Phys.* **86**, 253 (2014).

[2] Van de Walle, C. G., & Neugebauer, J. First-principles calculations for defects and impurities: Applications to III-nitrides. *J. Appl. Phys.* **95**, 3851 (2004).

4. The authors should evaluate the local strain at the interface between the Al_2Ca Laves phase and the surrounding Mg alloy host. For instance, Geometric Phase Analysis (GPA) calculations should be performed.

Response:

We thank the reviewer for this constructive suggestion. GPA is a powerful method for quantifying local strain fields associated with dislocation cores, grain boundaries, and related lattice distortions. We applied GPA to our aberration-corrected, high-resolution STEM images to quantitatively assess the interfacial strain between the confined 2D Al_2Ca Laves phase and the surrounding Mg-alloy matrix. The resulting strain maps (Figure R9) reveal tensile strain along the (0001) matrix planes adjacent to the precipitate, with peak values consistent with the atomistic volume strain (3–5%) predicted by our MD simulations (Figure 8a in revised manuscript).

It is important to emphasize, however, that unlike the large strain fields typically observed at dislocation cores or grain boundaries, the atomic-scale volume strain within the grain interior is highly sensitive to the quality of high-resolution imaging. Minor perturbations in the experimental environment—such as subtle mechanical vibrations, humidity fluctuations, or air flow—can degrade atomic-resolution imaging and thereby affect the fidelity of strain quantification. Moreover, in convergent-beam STEM imaging, electron-beam irradiation may rapidly relax small local strains, preventing their faithful representation in the recorded images. By contrast, the MD-optimized structural model is free from such environmental and instrumental artefacts, and thus provides an unambiguous and comprehensive picture of the strain fields surrounding the precipitate, as shown in the revised Figure 8a.

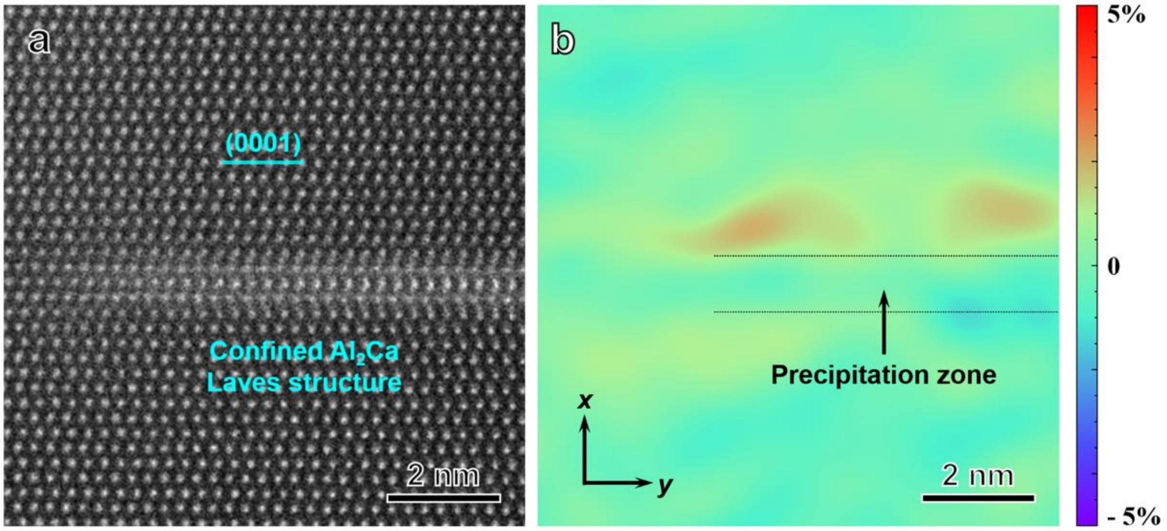

Figure R9. Atomic-scale characterization of lattice strain. (**a**) HAADF-STEM image acquired along the $[11\bar{2}0]_\alpha$ zone axis for the alloy aged at 200 °C for 24 h. (**b**) Corresponding geometric phase analysis (GPA) map of the ε_{xx} strain component, measured normal to the (0001) basal plane.

5. Is the size distribution of the precipitates influenced by annealing time, and does this subsequently affect the final hardness? Quantification of size evolution is lacking; only image data for 24 h and 500h are provided.

Response:

We thank the reviewer for the insightful assessment. In response, we performed additional atomic-scale characterization on samples aged for 8 h, 200 h, and 800 h (Figures. R10–R12). The results reveal a systematic evolution of the confined 2D Laves precipitate during ageing: its size increases from ~12 nm at 8 h to 30–50 nm at peak ageing (24 h), and with continued

over-ageing to 200 h, it further coarsens and stabilizes at 50-60 nm. As the ageing process advances, the elastic strain field at the precipitate–matrix interface progressively relaxes. The dissipation of this strain field reduces the diffusion barrier for Al atoms, promoting their sustained segregation to the interface and driving the thickening of the Laves phase along the $[0001]_{Mg}$ direction. In the 800 h over-aged sample, the precipitate has fully coarsened into a three-dimensional, block-like variant.

The hardness evolution of the alloy (Supplementary Figure 1) closely parallels these microstructural changes: hardness increases from the solution-treated state, reaches a peak of ~66 HV at peak ageing, and then declines during over-ageing. This trend reflects the competing contributions of precipitate size, number density and interfacial strain:

Before peak ageing, the rise in hardness is dominated by the rapidly increasing precipitate number density, which provides a high density of effective obstacles to dislocation motion.

After peak ageing, the decrease in hardness results from (i) precipitate coarsening (Ostwald ripening), which reduces their strengthening efficiency, and (ii) the relaxation and eventual disappearance of the interfacial elastic-strain field. The latter diminishes the coherency-strain contribution to strengthening and may accompany a transition to semi-coherent or incoherent interfaces that offer less resistance to dislocation glide.

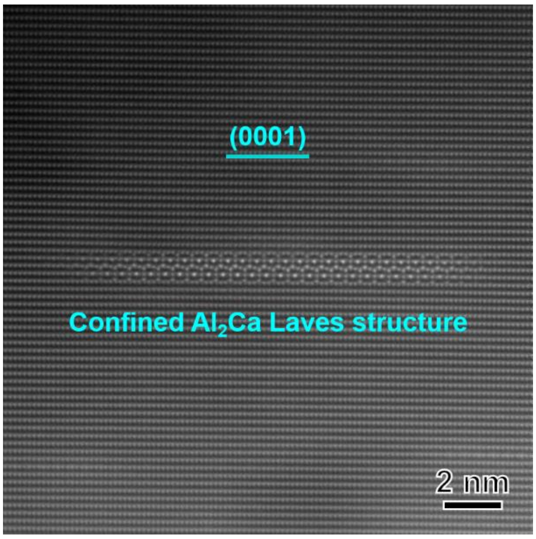

Figure R10. HAADF-STEM image reveals the presence of a confined 2D Laves precipitate in the Mg–2.0Al–1.0Ca alloy aged at 200 °C for 8 h. The image acquired along the $[1\bar{1}00]_{\alpha}$ zone axis.

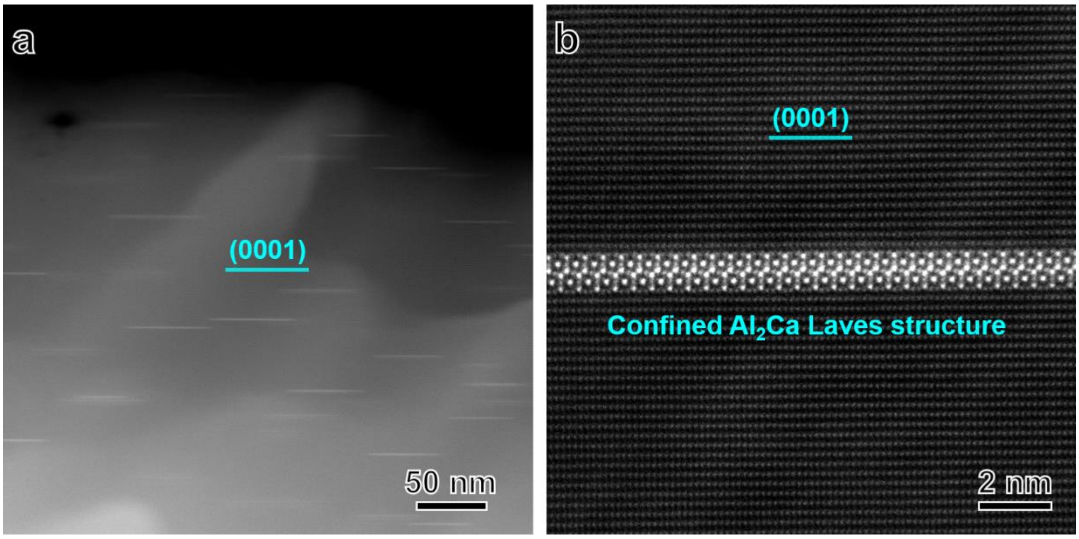

Figure R11. HAADF-STEM images reveal the presence of confined 2D Laves precipitates in the Mg–2.0Al–1.0Ca alloy aged at 200 °C for 200 h. The images acquired along the $[1\bar{1}00]_{\alpha}$ zone axis.

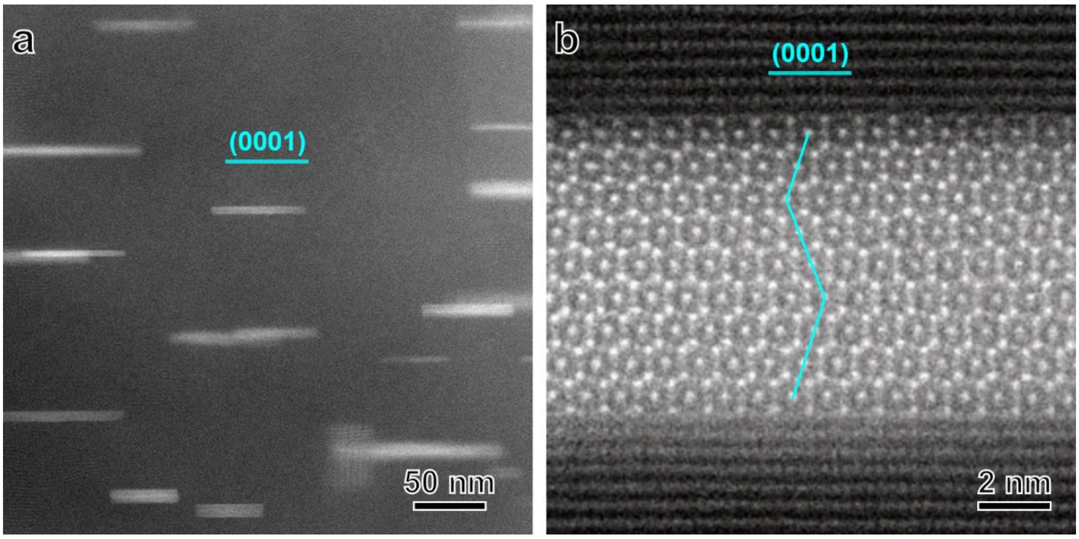

Figure R12. HAADF-STEM images reveal the presence of Laves precipitates in the Mg–2.0Al–1.0Ca alloy aged at 200 °C for 800 h. The images acquired along the $[1\bar{1}00]_{\alpha}$ zone axis.

In summary, the work primarily reports the discovery of a novel type of Al3Ca Laves phase but fails to provide a clear rationale for the main research objective. Although extensive first-principles calculations and MD simulations are presented, the construction and detailed analysis of the local coherent interface between the Al_2Ca Laves and the Mg matrix need careful consideration. This is particularly crucial for the interfacial transition region, as local strain critically impacts formation energy. Consequently, I cannot recommend the manuscript in its current version.

Response:

We sincerely thank the reviewer for their candid and constructive comments, which have

guided substantial improvements to the manuscript. We appreciate the reviewer's recognition of our theoretical calculations and have carefully addressed all concerns regarding the research objectives and interfacial analysis.

As noted in our earlier responses, the significance of this work extends beyond the identification of a previously unreported Al_2Ca phase. Crucially, it reveals that this confined 2D Laves structure represents a fundamental structural motif common to a broad class of Laves phases. Its discovery provides key insights into the nucleation pathways, stability, and structural evolution of such compounds. Our study resolves, at atomic resolution, how this confined 2D structural unit is embedded within the Mg matrix. Through first-principles calculations and MD simulations, we elucidate its nucleation pathway, growth behavior, and the physical origin of its resistance to coarsening.

Addressing the reviewer's specific interest in the interfacial transition region, we performed a systematic local strain analysis. GPA of atomic-resolution HAADF-STEM images (Figure R9) confirms the presence of tensile strain in the Mg matrix adjacent to the precipitate, in agreement with theoretical predictions. This strain field energetically disfavors the incorporation of the smaller Al atoms, thereby suppressing Al diffusion along the precipitate's longitudinal direction (see Fig. R1 and related responses). This mechanism is essential for stabilizing the confined 2D Laves structure. Furthermore, during extended annealing, the interfacial elastic strain gradually relaxes at elevated temperature. This relaxation lowers the diffusion barrier for Al, promotes its continued segregation to the interface, and consequently drives thickening of the Laves phase along the $[0001]_{Mg}$ direction. This process accounts for the structural evolution observed during long-term ageing.

Point-by-point response to the reviewers' comments

Reviewer #1

The authors have addressed all my concerns. I would like to recommend this manuscript to be published in Nature Communications.

Response:

We sincerely thank the reviewer for the careful evaluation of our manuscript and for the positive recommendation.

Reviewer #2

I would like to thank the authors for crafting a thoughtful, and detailed, response letter. They have adequately addressed all of my comments/concerns.

This manuscript is now suitable for publication in Nature Communications.

Response:

We greatly appreciate the reviewer's professional assessment and positive recommendation for publication in Nature Communications.

Reviewer #3

Thank you for the authors' detailed response. In the revised manuscript, the authors have supplemented the STEM experimental characterization and associated theoretical calculations. These additions strongly support the claim regarding the importance of the newly identified Al_2Ca Laves phase. All of my concerns have been

addressed satisfactorily, and I therefore recommend publication in Nature Communications.

Response:

We greatly appreciate the reviewer's professional assessment, constructive feedback, and positive recommendation for publication in Nature Communications.

Minor suggestion:

I suggest that the authors put the discussion of the Mg element in the EDS characterization of the Al_2Ca phase region presented in Fig. R8 in the main text. This would provide a more comprehensive understanding of the elemental analysis in this area.

Response:

We sincerely thank the reviewer for this thoughtful and constructive suggestion. We agree that discussing the Mg signal in the EDS characterization of the Al_2Ca phase region shown in Fig. R8 could provide additional context for the elemental analysis in this area.

However, after careful consideration, we have decided not to incorporate this discussion into the main text at this stage. The main reason is that this aspect is not central to the primary focus of the present study, and a detailed discussion of this issue would substantially lengthen the relevant section and may distract readers from the main scientific message of the manuscript.

Nevertheless, in accordance with the editorial and peer-review policy of *Nature Communications*, the reviewer comments and the authors' responses will be published alongside the paper. Therefore, readers interested in this specific issue will be able to access our detailed discussion and clarification in the peer-review file.

We are grateful to the reviewer for this professional and insightful comment, which has prompted us to further reflect on the presentation and focus of the manuscript.
